# Antimicrobial resistance interventions in Latin America and the Caribbean: a scoping review of reported interventions between 2018–2024

**DOI:** 10.1186/s13756-025-01629-z

**Published:** 2025-11-11

**Authors:** Ernesto Gozzer, Naysha Becerra-Chauca, Mohammed Abba-Aji, Veronika J. Wirtz, Gloria Cordoba, Fredy Canchihuamán, Rajeev Peeyush Nagassar, Samantha Yañez-Diaz, Penélope S. Brou, Carolina J. Delgado-Flores, Shaffi Fazaludeen Koya

**Affiliations:** 1 Facultad de Salud Pública y Administración, Universidad Peruana Cayetano Heredia, Lima, Peru; 2 School of Public Health, Boston University, Boston, USA; 3 School of Public Health, Washington University, St. Louis, USA; 4 International Centre for Antimicrobial Resistance Solutions, Copenhagen, Denmark; 5 The Sangre Grande Hospital, The Eastern Regional Health Authority, Sangre Grande, Trinidad and Tobago; 6 Health Innovation Laboratory, Institute of Tropical Medicine “Alexander von Humboldt”, Universidad Peruana Cayetano Heredia, Lima, Peru; 7 Grupo Peruano de Investigación en Medicamentos, Políticas y Servicios Farmacéuticos (GPIMPSF), Universidad Científica del Sur, Lima, Peru

**Keywords:** Drug resistance, AMR, Bacterial, Program evaluation, One health, Latin America, Review literature as topic

## Abstract

**Background:**

Antimicrobial resistance (AMR) is a critical global health challenge, linked to 4·71 million deaths in 2021 and affecting human health, animals, food, plants, and the environment. This scoping review aims to map out published interventions addressing AMR in the Latin America and Caribbean (LAC) region.

**Methods:**

We searched PubMed, Web of Science, LILACS, and grey literature for articles reporting the implementation of AMR programs, interventions, or policies aimed at tackling AMR published between January 2018 and December 2024.

**Results:**

A total of 82 studies were included, comprising 64 peer-reviewed articles and 18 from grey literature. The majority (*n* = 75) focused on human health, while a smaller subset (*n* = 7) addressed animal health. Geographically, most studies were conducted in Brazil (*n* = 32) and Colombia (*n* = 22) with only one study in the Caribbean. Antimicrobial stewardship interventions were the primary focus in 50 studies. Only 53 out of 74 studies included an evaluation of the intervention.

**Conclusion:**

Significant gaps remain in AMR research in LAC, particularly in animal and environmental health. Rigorous intervention evaluations are needed to generate high-quality evidence for policy and practice. Increased funding for intervention and implementation research across all sectors is crucial to tackling AMR regionally and globally.

**Supplementary Information:**

The online version contains supplementary material available at 10.1186/s13756-025-01629-z.

## Background

Antimicrobial resistance (AMR) is one of the greatest threats to global health security. Based on a Global Burden of Disease study, the number of human deaths linked to bacterial AMR in 2021 was estimated to be 4·71 million, of which 1·14 million were directly related to bacterial AMR [1]. Besides, the rise of AMR significantly impacts not only human but animal health, the agri-food system and the environment [2, 3]. Thus, the One Health approach, which highlights this interdependent and multisectoral nature of AMR is critical towards curbing the rise of AMR [4]. 

The One Health approach addresses human, animal, and environmental health together. This approach is reflected in the AMR Global Action Plan (GAP), launched in 2015 by three organizations: the World Health Organization (WHO), Food and Agriculture Organization (FAO), and World Organization for Animal Health (WOAH, formerly OIE). The plan urges countries to develop national action plans (NAP) [5]. In 2022, the United Nations Environment Program (UNEP) joined these three organizations, forming the Quadripartite. Since 2016, progress has been monitored annually through the Tracking Antimicrobial Resistance Country Self-Assessment Survey (TrACSS).

The Latin America and Caribbean region faces particularly severe AMR challenges that distinguish it from other global regions. The region experiences the world's highest AMR mortality burden relative to population, with 322,000 deaths in 2021 projected to reach 650,000 by 2050 [6]. LAC exhibits catastrophic resistance patterns, including the world's highest extended-spectrum β-lactamase prevalence at 30.3% and methicillin-resistant Staphylococcus aureus rates averaging 45%—significantly exceeding rates in Europe and North America [7]. Despite 30 of 33 countries having developed NAP aligned with WHO recommendations, implementation remains severely constrained by limited healthcare infrastructure, fragmented public–private healthcare systems, and cultural factors that promote self-medication rates of 14–26% across the region [8]. These unique regional characteristics—including healthcare systems with only 2.1 hospital beds per 1,000 population compared to 4.7 in OECD countries, widespread over-the-counter antibiotic access despite regulatory restrictions, and socioeconomic barriers that drive inappropriate antibiotic use—create AMR drivers fundamentally different from other global regions and necessitate tailored intervention approaches specific to the LAC context [8].

The Call-to-Action event on Antimicrobial Resistance (AMR) organized in November 2021 by the Welcome Trust with the support of several organizations, including the International Centre for Antimicrobial Resistance Solutions (ICARS), identified constraints on resource mobilization, non-operationalization of the One Health concept, lack of a formal AMR program, and data and evidence gaps as barriers to NAP implementation [9]. Informed by the varying levels of implementation reported by the TrACSS and the call to action to improve data and evidence on the implementation of AMR interventions, we set out to map out the existing literature on AMR interventions reported in the LAC region within the past five years.

Accordingly, we conducted a scoping review with the aim of mapping out the extent and range of published policies, interventions or programs (defined as a set of activities) implemented with an explicit intention to address AMR in the LAC region. A scoping review was found appropriate due to the broad nature of this topic and the usefulness of a scoping review in identifying gaps in existing research [10]. Within the context of AMR research in Latin America, this approach has been previously used to map evidence and identify research gaps in hospital antimicrobial stewardship and AMR transmission in rural communities [11, 12]. Beyond the LAC region, scoping reviews have been used in African countries to gain insights on the current state of national antimicrobial stewardship (AMS) activities and identify relevant actors in the region [13].

## Main text

### Methods

#### Search strategy and selection criteria

We conducted a systematic search in three databases—PubMed, Web of Science, and Latin America and the Caribbean Literature on Health Sciences (LILACS)—to identify articles published between January 1, 2018, and June, 2023. Additionally, we searched for gray literature (e.g., policy documents and reports) through relevant institutional websites. These included publications from the WHO, FAO, WOAH (formerly OIE), UNEP, United Nations International Children’s Fund (UNICEF), Pan American Health Organization (PAHO), Ministries or departments of health, veterinary, agriculture and environment in different LACs. Key persons from health, education and other ministries relevant to One Health (agriculture, environment, animal health, veterinary, etc.) were also contacted. To ensure the inclusion of the most recent evidence, we subsequently updated the search in PubMed only, extending the coverage through December 31, 2024. We included publications from the last seven years (1 Jan 2018—Dec 31 st, 2024). The search strategies are in the Appendix Table 1.

**Table 1:** Characteristics of evaluation studies and non-evaluation studies

	**Evaluation studies**	**non evaluation studies**	Total*
	***n* = 53**	***n* = 29**	***n* = 82**
Type of intervention			
Antimicrobial stewardship	38 (71.7%)	12 (41.4%)	50 (61%)
Surveillance	4 (7.5%)	13 (44.8%)	17 (20.7%)
Awareness	2 (3.8%)	1 (3.4%)	3 (3.7%)
Capacity Building	5 (9.4%)	1 (3.4%)	6 (7.3%)
Infection prevention and control	8 (15.1%)	6 (20.7%)	14 (17.1%)
Policy—regulations	2 (3.8%)	2 (6.9%)	4 (4.9%)
Sector			
Human Sector	52 (98.1%)	23 (79.3%)	75 (91.5%)
Animal Sector	1 (1.9%)	6 (20.7%)	7 (8.5%)
Environmental Sector	1 (1.9%)	2 (6.9%)	3 (3.7%)
One Health AMR Pilars			
Surveillance	2 (3·8%)	13 (44.8%)	15 (18.3%)
Intervention	42 (79.2%)	15 (51.7%)	57 (69.5%)
Transmission	1 (1·9%)	4 (13.8%)	5 (6.1%)
Behavioral Insights and Change	15 (28.3%)	7 (24.1%)	22 (26.8%)
Policy and Economics	2 (3.8%)	2 (6.9%)	4 (4.9%)
Type of source			
Academic article published in journal	53 (100%)	18 (62.1%)	71(86·6%)
Credible report (published in official web pages)	0 (0·0%)	11 (37.9%)	11 (13.4%)
Settings			
Healthcare facility	42 (79.2%)	17 (58.6%)	59 (72%)
Community	7 (13.2%)	3 (10.3%)	10 (12·2%)
Animal Farms	1 (1.9%)	5 (17.2%)	6 (7.3%)
Others (virtual environment, homecare, etc.)	2 (3.8%)	4 (13·8%)	6 (7.3%)
Not specified	1(1.9%)	0(0·0%)	1 (1·2%)

^*^Some interventions included more than one type of intervention, sector, or pilar

*Abbreviations*: *AMR* (Antimicrobial Resistance)

Our inclusion criteria required articles to report the implementation of a program, intervention, or policy (defined as a set of activities) explicitly intended to address AMR. Studies had to mention AMR or related terms (e.g., resistance to antibiotics) explicitly in their introduction, indicating that the intervention aimed to impact efforts against AMR, regardless of whether this was the primary objective of the study. Eligible interventions could address human, animal, or environmental health and were not restricted by scale. Additionally, articles needed to provide details on the activities implemented and specify the region or country of implementation. Any type of study (Observational, quasi experimental or experimental) was included.

We excluded studies on interventions restricted to only viral, parasitic or fungal resistance. We did not exclude articles based on language.

#### Selection process

**Fig. 1:**
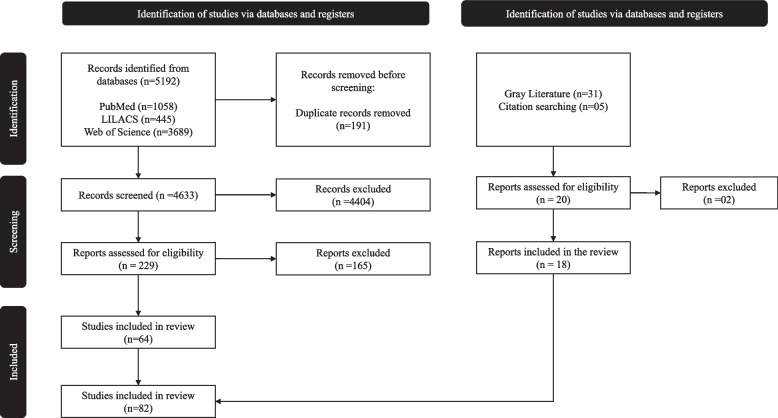
Preferred Reporting Items of Systematic Reviews (PRISMA) flow diagram

The article selection process was conducted in a three-stage process. The first stage was screening based on the title and abstract of the records identified from the three databases. This was conducted by four reviewers, NBC, PSB, SYD and CJDF. The second stage involved screening of full-text articles to assess eligibility for inclusion into the review based on the inclusion criteria mentioned above. The excluded studies are listed in Appendix Table 2. The third stage of the selection process was data extraction of the included studies using a standard Excel sheet (used by all four reviewers). Additionally, we complemented our database search with a gray literature search. The final search retrieved 5·192 records, all of which were retrieved and transferred to Rayyan [42], a collaborative systematic review tool. We identified 191 duplicates through an automatic detection tool. After deduplication, we were left with 4·633 records, for screening based on title and abstract. We identified 229 eligible articles which were assessed in full text, out of which 64 articles were included. We identified 18 additional records that met our inclusion criteria through gray literature search. Finally, 82 reports were selected for data extraction and included in the review (Fig. 1). We document our study selection flow in accordance with the Preferred Reporting Items of Scoping Reviews (PRISMA- ScR) guidelines in Fig. 1 [43].

**Table 2:** Description of non-evaluation studies

Nº	First author	Publication year	Source type	Type of intervention	Sector	One Health Pillars
1	Dias [14]	2021	Academic article	Surveillance	Human health	Integrated Surveillance
2	Silva [15]	2020	Academic article	Antimicrobial stewardship	Human health	Intervention
3	Cassettari [16]	2021	Academic article	Antimicrobial stewardship	Human health	Intervention
4	Valderrama-Rios [17]	2023	Academic article	Antimicrobial stewardship	Human health	Intervention, Behavioral Insights and Change
5	Oliveira [18]	2021	Academic article	Antibiotic waste manages	Environmental health	Intervention
6	Ketcherside [19]	2020	Academic article	Infection prevention and control, Antimicrobial stewardship	Human health	Intervention, Behavioral Insights and Change
7	Londoño-Ruiz [20]	2023	Academic article	Surveillance, Antimicrobial stewardship	Human health	Integrated Surveillance, Interventions,
8	Ramon Pardo [21]	2018	Academic article	Surveillance	Human health	Integrated Surveillance
9	Servicio Nacional de Pesca y Acuicultura [22]	2022	Credible reports	Antimicrobial stewardship	Animal health	Intervention
10	Servicio Nacional de Pesca y Acuicultura [22]	2023	Credible reports	Surveillance	Animal health	Integrated Surveillance
11	Hospital de Clínicas "Dr. Manuel Quintela" [23]	2018	Credible reports	Antimicrobial stewardship	Human health	Intervention
12	Programa de Apoio ao Desenvolvimento Institucional do Sistema Único de Saúde [24]	Not specified	Credible reports	Surveillance Awareness	Human health	Integrated Surveillance
13	Pillonetto [25]	2021	Academic article	Surveillance	Human health	Integrated Surveillance
14	D'Or Institute for Research and Education [26]	2022	Credible reports	Antimicrobial stewardship	Human health	Interventions, Behavioral Insights and Change
15	International Centre for Antimicrobial Resistance Solutions [27]	Not specified	Credible reports	Infection prevention and control	Animal health	Interventions, Behavioral Insights and Change
16	International Centre for Antimicrobial Resistance Solutions [28]	Not specified	Credible reports	Infection prevention and control	Human health, Animal health, environment health	Transmission, Behavioral Insights and Change
17	International Centre for Antimicrobial Resistance Solutions [29]	Not specified	Credible reports	Infection prevention and control	Animal health	Transmission, Behavioral Insights and Change
18	International Centre for Antimicrobial Resistance Solutions [30]	Not specified	Credible reports	Infection prevention and control	Animal health	Transmission, Behavioral Insights and Change, Intervention
19	Tomazini [31]	2022	Academic article	Surveillance	Human health	Integrated surveillance
20	Castro-Espinosa [32]	2022	Academic article	Surveillance, Antimicrobial stewardship	Human health	Integrated surveillance
21	Gorla [33]	2018	Academic article	Surveillance	Human health	Integrated surveillance
22	Rodríguez [34]	2023	Academic article	Policy—Regulation	Human health	Economics and policy
23	Campos-Lara [35]	2021	Academic article	Antimicrobial stewardship	Human health	Intervention
24	Kekre [36]	2021	Academic article	Surveillance	Human health	Integrated surveillance, Interventions
25	Holguín [37]	2020	Academic article	Antimicrobial stewardship	Human health	Intervention
26	Pfaller [38]	2019	Academic article	Surveillance	Human health	Integrated surveillance
27	World Health Organization [39]	2022	Credible reports	Surveillance	Human health	Integrated surveillance
28	Food and Agriculture Organization [40]	2023	Credible reports	Policy – Regulation, Capacity building, Surveillance, Antimicrobial stewardship	Human health	Economics and Policy, Interventions, Integrated surveillance
29	Fabre [41]	2024	Academic article	Infection, prevention and control	Human health	Transmission

#### Data charting

Using the standardized data extraction sheet, we collected information on the following variables: year, title, country, language, implementation period, area of implementation, study design, settings, interventions implemented, and outcomes evaluated.

#### Synthesis of the results

We summarized the findings in terms of country of study, publication year, thematic area, and type of study design. We used a descriptive‐analytical approach, a commonly used framework to synthesize and summarize some of the key findings. Additionally, we classified the outcomes based on the pillars of the One Health Priority Research Agenda framework, developed by the Quadripartite, which focuses on human, animal, environmental, and ecosystem health [44]. We present the results using tables and figures.

## Results

### Overview of included studies

**Fig. 2:**
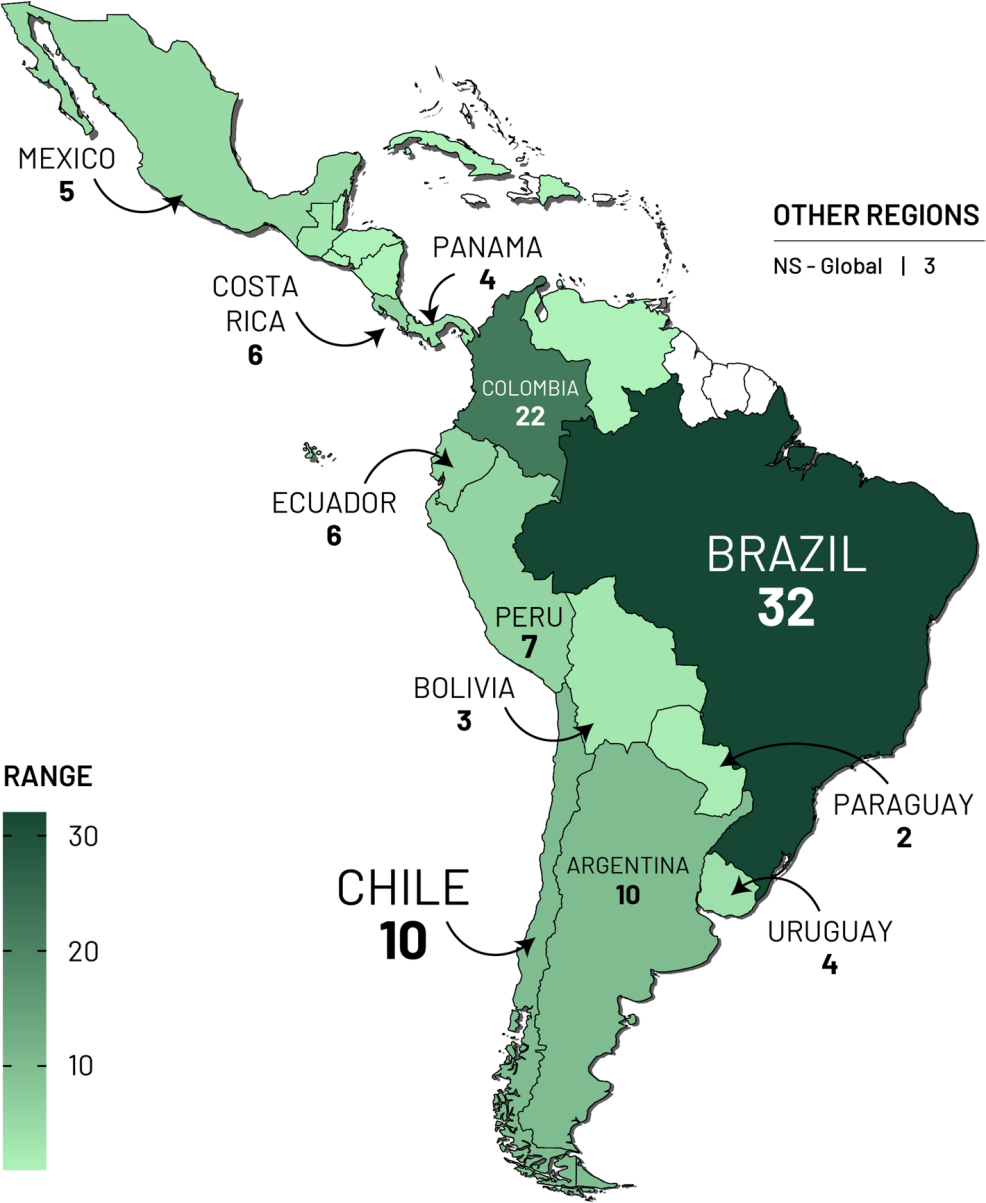
Geographical distribution of included studies

We identified 82 studies that met the inclusion criteria, comprising interventions, programs, or policies specifically designed to address antimicrobial resistance across human, animal, agricultural, and environmental health sectors. The geographical distribution of these initiatives across the LAC region revealed a complex and uneven landscape of AMR-related activities. Brazil contributed the highest number of studies (*n* = 32), followed by Colombia (*n* = 22) and Argentina Chile with ten studies each (Fig. 2). Conversely, no studies were identified from Guyana, French Guiana, or Suriname. The Caribbean region was particularly underrepresented, with only a single study originating from Guadeloupe. (Fig. 2).

Of the 82 studies analyzed, 53 included an evaluation of the outcomes or impacts of the implemented intervention (“evaluation studies”), while 29 provided no assessment data (“non-evaluation studies”). Most studies focused on the human health sector, while only one study comprehensively addressed all core components of the One Health AMR sectors: human, animal, and environmental health. Among “evaluation studies”, about 43% (*n* = 23) used a quasi-experimental study design, with less than 10% (*n* = 4) using an experimental design. (Table 1).

The following sections provide a detailed overview of the characteristics of included articles, grouped according to whether or not they included evaluation.

## Non evaluation studies (*n* = 29)

### One health sectors

A large number of studies (23/29) focused on human health, addressing various aspects of AMR through interventions in hospital settings, community health, and healthcare policy. Five articles reported on the animal sector and one study focused only on environmental health. Only one article adopted a multisectoral approach, addressing human health, animal health, and environmental health [29].

### One health AMR pillars

Based on the One Health AMR pillar framework—Transmission, Integrated Surveillance, Interventions, Behavioral Insights and Change and Economics and Policy— most of the included articles were in the interventions (*n* = 15) and surveillance pillars (*n* = 13). Seven studies emphasized behavioral insights and change aimed at changing behaviors and practices related to antimicrobial use. Fewer studies focused on transmission (*n* = 4) and economic and policy measures (*n* = 2).

### Types of interventions

Twelve studies focused on antimicrobial stewardship, implementing various measures to optimize antimicrobial use. Surveillance activities were covered in 13 studies, which implemented monitoring systems for AMR. Infection prevention and control (IPC) was addressed in six studies mainly through hygiene protocols and environmental measures. Two studies focused on policy and regulation interventions, implementing regulatory measures for antimicrobial use. One study focused on raising awareness about AMR, while another addressed capacity building through educational and training programs.

A detailed description of the Sectors, One Health AMR Pillar, and types of interventions for these studies is at Table 2.

## Evaluation studies (*n* = 53)

### One health sectors

Almost all studies (*n* = 52) focused on human health, addressing various aspects of AMR through interventions in hospital settings, community health, and healthcare policy. Only one study addressed AMR in the animal health sector, focusing on the effect of antibiotics in livestock feed [45]. Similarly, only one study [46], which assessed the effectiveness of filtration system in removing resistant bacteria from water for human consumption, focused on environmental and human health. A comprehensive description of the included studies with intervention evaluation is at Table 3.

**Table 3:** Description of evaluation studies

	**First author**	**Publication year**	**Source type**	**Type of intervention**	**Sector**	**One Health Pillars**	**Type of study**
1	Zequinao [47]	2020	Academic article	Antimicrobial stewardship	Human health	Intervention	Quasi experimental
2	Quirós [48]	2022	Academic article	Antimicrobial stewardship	Human health	Intervention	Quasi experimental
3	Hernández-Gómez [49]	2019	Academic article	Antimicrobial stewardship	Human health	Intervention	Quasi experimental
4	Dos Santos [50]	2019	Academic article	Antimicrobial stewardship	Human health	Intervention	Quasi experimental
5	Doltrario [51]	2022	Academic article	Antimicrobial stewardship	Human health	Intervention	Quasi experimental
6	Ávila [52]	2019	Academic article	Antimicrobial stewardship	Human health	Intervention	Quasi experimental
7	Holguín [53]	2021	Academic article	Antimicrobial stewardship	Human health	Intervention	Quasi experimental
8	Laks [54]	2019	Academic article	Education—capacity Building	Human health	Behavioral Insights and Change	Quasi experimental
9	Borges [55]	2020	Academic article	Infection prevention and control	Human health	Intervention	Experimental
10	Brandileone [56]	2021	Academic article	Infection prevention and control	Human health	Intervention, Transmission	Observational
11	Moura [57]	2022	Academic article	Policy—Regulation	Human health	Behavioral Insights and Change, Economics and Policy	Not specified
12	Tutida [45]	2021	Academic article	Infection prevention and control	Animal health	Intervention	Experimental
13	Zarpellon [58]	2018	Academic article	Surveillance	Human health	Integrated Surveillance Behavioral Insights and Change	Observational
14	Jorge [59]	2020	Academic article	Antimicrobial stewardship	Human health	Intervention	Quasi experimental
15	De la Rosa-Zamboni [60]	2018	Academic article	Infection prevention and control Antimicrobial stewardship	Human health	Intervention, Behavioral Insights and Change	Observational
16	Bastidas-Caldes [46]	2022	Academic article	Infection prevention and control	Environmental health, Human health	Intervention, Transmission	Not specified
17	Chávez [61]	2021	Academic article	Antimicrobial stewardship	Human health	Intervention	Observational
18	Rojas-Bonilla [62]	2020	Academic article	Antimicrobial stewardship	Human health	Intervention	Quasi experimental
19	Pérez-Lazo [63]	2023	Academic article	Surveillance Antimicrobial stewardship	Human health	Intervention	Quasi experimental
20	Valderrama-Rios [64]	2023	Academic article	Antimicrobial stewardship	Human health	Intervention, Behavioral Insights and Change	Quasi experimental
21	Diaz-Madriz [65]	2022	Academic article	Antimicrobial stewardship	Human health	Intervention	Observational
22	Vargas [66]	2022	Academic article	Surveillance	Human health	Integrated surveillance, Intervention	Experimental
23	Pallares [67]	2022	Academic article	Antimicrobial stewardship	Human health	Intervention	Observational
24	Oliveira da Silva [68]	2022	Academic article	Antimicrobial stewardship	Human health	Intervention	Observational
25	Staneloni [69]	2022	Academic article	Infection prevention and control	Human health	Behavioral Insights and Change	Quasi experimental
26	Jorge [70]	2022	Academic article	Antimicrobial stewardship	Human health	Intervention	Quasi experimental
27	Romo-Castillo [71]	2022	Academic article	Antimicrobial stewardship	Human health	Intervention	Observational
28	Le Terrier [72]	2021	Academic article	Antimicrobial stewardship	Human health	Intervention	Observational
29	Rodrigues [73]	2021	Academic article	Antimicrobial stewardship	Human health	Surveillance, Intervention	Experimental
30	Paillier-González [74]	2021	Academic article	Antimicrobial stewardship	Human health	Interventions	Quasi experimental
31	Sanchez [75]	2021	Academic article	Awareness	Human health	Behavioral Insights and Change	Quasi experimental
32	Abrudan [76]	2021	Academic article	Education—capacity Building	Human health	Behavioral Insights and Change	Quasi experimental
33	Díaz-Madriz [77]	2020	Academic article	Antimicrobial stewardship	Human health	Intervention	Observational
34	Sosa-Hernández [78]	2020	Academic article	Antimicrobial stewardship	Human health	Intervention	Observational
35	Araujo da Silva [79]	2020	Academic article	Antimicrobial stewardship	Human health	Intervention	Quasi experimental
36	Mazzillo [80]	2020	Academic article	Infection prevention and control, Antimicrobial stewardship	Human health	Intervention	Observational
37	Cruz [81]	2019	Academic article	Antimicrobial stewardship	Human health	Intervention, Behavioral Insights and Change	Quasi experimental
38	Moreira da Costa [82]	2019	Academic article	Policy—Regulation	Human health	Integrated surveillance	Quasi experimental
39	Antonello [83]	2019	Academic article	Education—capacity Building	Human health	Intervention	Observational
40	Fica [84]	2018	Academic article	Antimicrobial stewardship	Human health	Intervention	Observational
41	Angarita-Díaz [85]	2022	Academic article	Awareness, Education—capacity Building	Human health	Behavioral Insights and Change	Quasi experimental
42	Silva [86]	2022	Academic article	Antimicrobial stewardship	Human health	Behavioral Insights and Change	Observational
43	Quiros [87]	2022	Academic article	Antimicrobial stewardship	Human health	Intervention, Behavioral Insights and Change	Observational
44	Molano [88]	2018	Academic article	Antimicrobial stewardship	Human health	Intervention, Behavioral Insights and Change	Observational
45	Pallares [89]	2023	Academic article	Antimicrobial stewardship	Human health	Intervention, Behavioral Insights and Change	Observational
46	Hernández-Gómez [49]	2019	Academic article	Antimicrobial stewardship	Human health	Intervention, Behavioral Insights and Change	Observational
47	Cornistein [90]	2023	Academic article	Infection, prevention and control, Antimicrobial stewardship and Surveillance	Human health	Intervention	Observational
48	Custódio [91]	2024	Academic article	Antimicrobial stewardship	Human health	Intervention	Quasi experimental
49	Bispo da Silva [92]	2024	Academic article	Antimicrobial stewardship	Human health	Intervention	Observational
50	Díaz-Madriz [93]	2023	Academic article	Antimicrobial stewardship	Human health	Intervention	Quasi experimental
51	Seas [94]	2024	Academic article	Antimicrobial stewardship	Human health	Intervention	Observational
52	Neves Giovanetti [95]	2024	Academic article	Antimicrobial stewardship, Capacity building	Human health	Intervention	Observational
53	Rojop [96]	2024	Academic article	Policy regulation	Human health	Policy and economics	Observational

### One health AMR pillars

Based on the One Health AMR pillar framework, most studies (*n* = 42) fell under the “Intervention pillar”. This was followed by studies that fell under behavioral insights and change that aim to alter behaviors and practices related to antimicrobial use (*n* = 15). Very few studies addressed integrated surveillance (*n* = 2), economic and policy measures (*n* = 2), or transmission (*n* = 1).

### Types of interventions

Regarding the types of evaluated interventions, there was a heavy focus on AMS (*n* = 38) with a significant number of studies, implementing various interventions aimed at optimizing antimicrobial use through prescription restrictions, audits, telemedicine, rapid diagnostics, and educational feedback. IPC was addressed in seven studies (*n* = 8) through hygiene protocols, environmental measures, vaccination programs, and hand hygiene practices. Five studies addressed capacity building through educational and training programs to improve knowledge and practices. Surveillance activities were covered in four studies, which implemented systems to monitor antimicrobial resistance and infection rates. Three studies were on policy and regulation interventions, implementing regulatory measures to control antimicrobial use. While only two studies each focused on raising awareness about AMR.

### Impact outcomes reported by articles with evaluation

The outcomes assessed for the interventions varied widely across the studies, reflecting the diverse approaches and contexts in which they were implemented. A detailed description of the activities and outcomes are reported in Table 4.

**Table 4:** Activities and outcomes reported by evaluation studies

	**First author**	**Title of Study**	**Setting**	**Activities of intervention**	**Outcomes evaluated**
1	Zequinao [47]	A broad-spectrum beta-lactam-sparing stewardship program in a middle-income country public Hospital: antibiotic use and expenditure outcomes and antimicrobial susceptibility profiles	Healthcare facility	Prescription restriction, Guideline implementation, Antimicrobial prescription audit/feedback	Antimicrobial consumption, ATM-related costs
2	Quirós [48]	Antimicrobial stewardship programs in adult intensive care units in Latin America: Implementation, assessments, and impact on outcomes	Healthcare facility	No specified since every country implemented different measurements	Antimicrobial consumption, Appropriateness of antimicrobial treatments, Crude mortality, Multidrug-resistant microorganisms in healthcare-associated infections (MDRO-HAIs)
3	Hernández-Gámez [49]	Antimicrobial Stewardship programs in Peru: A fundamental agreement]	Healthcare facility	Rapid diagnosis, Prescription restriction, Antimicrobial consumption monitoring	Antimicrobial infection rate, Antimicrobial consumption
4	Dos Santos [50]	Antimicrobial stewardship through telemedicine and its impact on multi-drug resistance	Healthcare facility	Immediate post-prescription reviews Antimicrobial prescription audit/feedback through Telemedicine tools	Antimicrobial consumption, Appropriateness of antimicrobial treatments, Antimicrobial resistance rate, Alcohol-based hand rub consumption, Time to ID consultation
5	Doltrario [51]	Assessment of preauthorization and 24-h expert consultation as a restrictive antimicrobial stewardship bundle in a Brazilian tertiary-care Hospital: an interrupted time series analysis	Healthcare facility	Antimicrobial prescription audit/feedback	Antimicrobial consumption
6	Ávila [52]	Cambios en las prescripciones y el consumo de antimicrobianos, luego de la implementación de recomendaciones de uso: experiencia en un Hospital universitario	Healthcare facility	Guideline implementation	Antimicrobial consumption, Appropriateness of antimicrobial treatments, Hospitalization length, Treatment length
7	Holguín [53]	Contribución del químico farmacéutico en los programas de gerenciamiento de antimicrobianos: estudio de cohortes ambispectivo	Healthcare facility	Antimicrobial prescription audit/feedback	Antimicrobial consumption, Treatment length, Costs
8	Laks [54]	Distance learning in antimicrobial stewardship: innovation in medical education	Other	Antimicrobial resistance courses	Knowledge gain
9	Borges [55]	Duration of antibiotic therapy in critically ill patients: a randomized controlled trial of a clinical and C-reactive protein-based protocol versus an evidence-based best practice strategy without biomarkers	Healthcare facility	Application of decision flowchart based on C-reactive protein	Treatment length
10	Brandileone [56]	Dynamics of antimicrobial resistance of Streptococcus pneumoniae following PCV10 introduction in Brazil: Nationwide surveillance from 2007 to 2019	Community	Pneumococcal Conjugate Vaccine immunization	Antimicrobial resistance of bacteria
11	Moura [57]	Effect on Antimicrobial Resistance of a Policy Restricting Over-the-Counter Antimicrobial Sales in a Large Metropolitan Area, São Paulo, Brazil	Community	Sale restriction policy	Antimicrobial resistance of bacteria
12	Tutida [45]	Effects of in feed removal of antimicrobials in comparison to other prophylactic alternatives in growing and finishing pigs	Animal Farms	Feeding without or with alternatives to antibiotic	Production índices. Economical performance, Sanitary performances
13	Zarpellon [58]	Epidemiologic surveillance of multidrug-resistant bacteria in a teaching Hospital: A 3-year experience	Healthcare facility	Guideline implementation, educational campaigns on hand hygiene, Isolation of patients infected, Surveillance of culture	Antimicrobial resistance, Infection rate
14	Jorge [59]	Evaluación de la implementación de un Programa de Uso Optimizado de Antimicrobianos en la pandemia de COVID-19	Healthcare facility	Guideline implementation	Antimicrobial consumption, Cost
15	De la Rosa-Zamboni [60]	Everybody hands-on to avoid ESKAPE: effect of sustained hand hygiene compliance on healthcare-associated infections and multidrug resistance in a pediatric Hospital	Healthcare facility	Education programs, Monthly feedback, Hand washing reminders, Hand hygiene monitoring, Surveillance Healthcare associated infections (HCAI)	Hand hygiene adherence, HCAIs infection rate, Central line-associated bloodstream infection
16	Bastidas-Caldes [46]	Removal of Extended-Spectrum Beta-Lactamase-Producing Escherichia coli, ST98, in Water for Human Consumption by Black Ceramic Water Filters in Low-Income Ecuadorian Highlands	Community	Detect bacterial pathogens in water, BCWF (black ceramic water filters) filtering installation	Bacterial pathogens present in water before and after BCWF installation
17	Chávez [61]	Impacto del monitoreo terapéutico de vancomicina y amikacina en la optimización de dosis de antimicrobianos en pacientes pediátricos	Healthcare facility	Monitoring of antimicrobial therapy, Dose adjustment	Percentage of reached the therapeutic range—initial doce, Required adjustment rate
18	Rojas-Bonilla [62]	Impact of an antimicrobial stewardship program in a pediatric third level Hospital in Panama	Healthcare facility	Restriction of antimicrobials, education of prescribers, Implementation of guidelines and protocols for antimicrobial use	Antibiotic consumption pre and post-intervention, Reduction in costs
19	Pérez-Lazo [63]	Impact of Adding a Rapid PCR-Based Blood Culture Identification Panel to the Antimicrobial Stewardship Program of Patients with Febrile Neutropenia in a Peruvian Referral Hospital	Healthcare facility	Rapid PCR-Based Blood Culture Identification Panel	Antibiotic resistance profile, Time to effective therapy, Relapse of bacteremia, In-Hospital mortality, 30-day-all-cause Hospital readmission
20	Valderrama-Rios [64]	Interventions to Improve Antibiotic Use in Hospitals with Different Levels of Complexity in Colombia: Findings from a Before-and-After Study and Suggestions for the Future	Healthcare facility	Guideline implementation, ASP using telemedicine	Adherence to the antibiotic recommendations, Antibiotic consumption
21	Diaz-Madriz [65]	Impact of a pharmacist-driven antimicrobial stewardship program on the prescription of antibiotics by intensive care physicians in a Latin American Hospital: A retrospective study	Healthcare facility	Pharmacy-driven antimicrobial stewardship program	Antibiotic consumption, Percentage of bacterial resistance
22	Vargas [66]	Impact of an active surveillance program and infection control measures on the incidence of carbapenem-resistant gram-negative bacilli in an intensive care unit	Healthcare facility	Data collection on carbapenem-resistant gram-negative bacilli (CRGNB)	Reduction of colonization CRGNB
23	Pallares [67]	Impact of antimicrobial stewardship programs on antibiotic consumption and antimicrobial resistance in four Colombian healthcare institutions	Healthcare facility	Optimizing antimicrobial usage	Antibiotic consumption
24	Oliveira da Silva [68]	The impact of monitoring software on antimicrobial management in a pediatric intensive care unit	Healthcare facility	Monitoring software	Antibiotic consumption, Percentage of bacterial resistance
25	Staneloni [69]	Program for the prevention of Carbapenemase-Producing Enterobacteria in critical units in Argentina during the COVID-19 pandemic	Healthcare facility	Hand hygiene, Environmental hygiene, Periodic surveillance with rectal swabs	Adherence to hand hygiene, Adherence to environmental hygiene, Adherence to Klebsiella pneumoniae carbapenemase (KPC) surveillance, Rate colonization KPC
26	Jorge [70]	Programa de optimización del uso de antimicrobiaNos durante la pandemia de COVID-19	Healthcare facility	Optimizing antimicrobial usage	Antibiotic consumption, UCI transfer. Mortality
27	Romo-Castillo [71]	Towards implementing an antibiotic stewardship programme (ASP) in Ecuador: evaluating antibiotic consumption and the impact of an ASP in a tertiary Hospital according to World Health Organization (WHO) recommendations	Healthcare facility	Restrictive measures on carbapenem dispensing, Use with pre-authorization	Antibiotic consumption
28	Le Terrier [72]	Impact of a restrictive antibiotic policy on the acquisition of extended-spectrum beta-lactamase-producing Enterobacteriaceae in an endemic region: a before-and-after, propensity-matched cohort study in a Caribbean intensive care unit	Healthcare facility	Restricted antibiotic policy, Monitoring extended-spectrum beta-lactamase-producing Enterobacteriaceae (ESBL-E)	Antibiotic use rate, ESBL-E infections, ESBL-E bacteremia, Duration of mechanical ventilation, Relapse or recurrence of sepsis during ICU stay, All-cause ICU mortality, All-cause Hospital mortality, ICU length of stay, Hospital length of stay, Patients who did not receive antibiotic therapy
29	Rodrigues [73]	Impact of restriction of over-the-counter sales of antimicrobials on antimicrobial resistance in Escherichia coli from community-onset urinary tract infections in inner Sao Paulo State, Brazil	Community	ASP and restriction of over-the-counter sales, Monitoring E. coli	Antimicrobial resistance profile
30	Paillier-González [74]	Prescripción antibiótica de los médicos generales: impacto de la evaluación y retroalimentación en un Hospital de tercer nivel en la ciudad de Medellín	Healthcare facility	Audit of antibiotic prescriptions, Feedback to prescribing doctors	Antibiotic consumption, Percentage feedbacks
31	Sanchez [75]	Reducing unnecessary antibiotic prescription through implementation of a clinical guideline on self-limiting respiratory tract infections	Healthcare facility	Educational sessions, Delivery of clinical guidelines	Antibiotic prescription rate, Appropriate antibiotic prescription rate
32	Abrudan [76]	Train-the-Trainer as an Effective Approach to Building Global Networks of Experts in Genomic Surveillance of Antimicrobial Resistance (AMR)	Not specified	Train-the-Trainer course integrating pedagogical aspects with genomic and bioinformatics activities	Training Course Design, Percentage of initiated training programs
33	Díaz-Madriz [77]	Impact of a pharmacist-driven antimicrobial stewardship program in a private Hospital in Costa Rica	Healthcare facility	Pharmacy-driven antimicrobial stewardship program	Antibiotic Consumption
34	Sosa-Hernández [78]	[Results of the Program for the Rational Use of Antimicrobials in a Mexican Hospital, 2013–2018]	Healthcare facility	Optimizing antimicrobial usage	Antibiotic Consumption, Costs
35	Araujo da Silva [79]	Use of the World Health Organization Access, Watch, and Reserve Classification to Follow Trends in Prescription of Antibiotic Use in Two Pediatric Intensive Care Units in Rio de Janeiro, Brazil	Healthcare facility	Use of WHO AWARE classification in ASP	Antibiotic consumption, Antibiotic consumption by AWARE
36	Mazzillo [80]	Uso racional de antibióticos y tecnología FilmArray para identificación rápida de bacteriemias en unidad de cuidados intensivos pediátrica	Healthcare facility	Implementation of FilmArray Blood Culture Identification and Urinary Tract Infection program	Microbial identification time, Duration of appropriate therapy, Antibiotic de-escalation
37	Cruz [81]	Impacto en el consumo de amikacina y ceftriaxone en una unidad de emergencias de adultos, luego de la implementación de una guía para el tratamiento de la infección urinaria alta	Healthcare facility	Guideline implementation via WhatsApp and print	Antibiotic Consumption, Sensitivity of E.coli, K. pneumonia and P.mirabilis
38	Moreira da Costa [82]	Restrictive measure for the commercialization of antimicrobials in Brazil: results achieved	Community	Implementation of ANVISA's restrictive commercialization of antimicrobials	Risk of hospital infection, Prevalence of antimicrobial resistance
39	Antonello [83]	The Use of Mobile Educational Tools to Improve Antimicrobial Prescription for the Treatment of Acute Pyelonephritis in Pregnancy: A Retrospective Cross-sectional Study	Healthcare facility	Introduction of institutional protocol and smartphone app	Antibiotic Prescription
40	Fica [84]	Long-term impact of competitive biddings and an antimicrobial stewardship program in a general Hospital in Chile	Healthcare facility	Competitive bidding process and ASP	Antibiotic Consumption (DDD), Cost, Global mortality, Infectious disease-associated mortality
41	Angarita-Díaz [85]	Impact of a virtual learning environment on the conscious prescription of antibiotics among Colombian dentists	Other	Educational strategy (virtual learning environments (VLEs))	Levels of awareness, attitudes, and intention to practice antibiotic prescription
42	Silva [86]	Uso de antibacterianos em gestantes antes e após regulamentação No Brasil: coortes de nascimentos de Pelotas, Rio Grande Do Sul, de 2004 e 2015	Community	Commercialization restriction	Antimicrobial consumption
43	Quiros [87]	1765. Antibiotic Consumption in a Healthcare System in Bolivia During the First Wave of COVID-19 Pandemic	Healthcare facility	Guideline implementation	Antimicrobial consumption (DDD)
44	Molano [88]	Implementation of a rational use of antibiotics program in an intensive care unit: Can outcomes be improved?	Healthcare facility	Analysis and feedback in real time, Educational strategies	Adequate antibiotic prescription, Length of Hospital stay, Antimicrobial consumption
45	Pallares [89]	Antimicrobial stewardship programs in seven Latin American countries: facing the challenges	Healthcare facility	Implemented customized on-site training to improve ASP development indicators, Implementation of updated antimicrobial guidelines, Education and training intervention	ASP-development and implementation of interventions, ASP education and training, Monitor and communicate ASP program progress, ASP core team member roles and responsibilities
46	Hernández-Gómez [49]	Impacto sobre la resistencia bacteriana de la revisión previa de la prescripción de antibióticos por el servicio farmacéutico en Hospitales del Atlántico(Colombia)	Healthcare facility	Antimicrobial prescription audit/Feedback	Antimicrobial resistance
47	Cornistein [90]	Synergy between infection control and antimicrobial stewardship programs to control carbapenem-resistant Enterobacterales	Healthcare facility	Healthcare-associated infection surveillance, hygiene activities compliance monitoring, education, feedback	CRE colonization and infection rates, hand hygiene adherence, antimicrobial consumption, and device-associated infection rates
48	Custódio [91]	Antibiotic stewardship and nosocomial infection prevention in critically ill patients: a quality improvement program	Healthcare facility	Guideline and checklist implementation, training on hygiene activities	Antimicrobial consumption, Healthcare-associated infection rate
49	Bispo da Silva [92]	Pharmacist-led antimicrobial stewardship program in the treatment of Staphylococcus aureus bacteraemia in paediatric patients: a multivariate analysis."	Healthcare facility	The management of antimicrobial use is mainly the responsibility of the clinical pharmacist, coordinated by the infectious diseases’ physician (program leader) and senior clinical pharmacist (co-leader)	Infection-related mortality and re-infection within 90 days
50	Díaz-Madriz [93]	Impact of the Five-Year Intervention of an Antimicrobial Stewardship Program on the Optimal Selection of Surgical Prophylaxis in a Hospital without Antibiotic Prescription Restrictions in Costa Rica: A Retrospective Study	Healthcare facility	Guideline implementation, Education and training intervention	Proportion of the selection of optimal antibiotics, Antibiotic consumption, Adverse events related to medication
51	Seas [94]	Implementing an Antimicrobial Stewardship Program in an Oncology Center in Lima, Peru: A Model for Low- and Middle-Income Countries	Healthcare facility	Guideline implementation, Education and training, Prescription audit/Feedback, Pharmacy interventions	Antimicrobial consumption, antimicrobial prescription, microbiological profile
52	Neves Giovanetti [95]	Eleven years impact of a stepwise educational program on healthcare associated infections and antibiotics consumption in an intensive care unit of a tertiary hospital in Brazil	Healthcare facility	Continuing education program, data collection system,	Healthcare associated infection, Hand hygiene adhesion, Sepsis diagnosis, Estimated costs
53	Rojop [96]	Informal sale of antibiotics in Guatemalan convenience stores before and after implementation of federal antibiotic dispensing legislation	Community	Ministerial Agreement 181–2019 requiring a prescription for antibiotics in pharmacies	Proportion of stores selling antibiotics (overall and by type), changes in stock before/after regulation; store-level changes in sale status

*Abbreviations*: *AMR* Antimicrobial Resistance, *ASP* Antimicrobial Stewardship Program, *AWARE* Access, Watch, Reserve (classification of antibiotic consumption by the WHO), *ANVISA* Agência Nacional de Vigilância Sanitária, *DDD* Defined Daily Dose, *WHO* World Health Organization

Many studies focused on AMS, with common outcomes including: a) reductions in antimicrobial consumption [47, 49–53, 59, 62, 64, 70], b) improved appropriateness of antimicrobial treatments [48, 50], and c) cost reductions related to antimicrobial use [47, 62]. Some studies also reported on the impact on antimicrobial resistance profiles [47, 64], infection rates [49], and other health outcomes such as crude mortality and multidrug-resistant microorganisms in healthcare-associated infections [48].

Several studies addressed IPC measures, reporting outcomes such as treatment length [55], antimicrobial resistance of bacteria [56, 57], production metrics and economic performance [45], adherence to hand hygiene and environmental hygiene protocols [69], and reduction in bacterial pathogens presence in water [46].

Surveillance activities focused on monitoring AMR and infection rates, with studies reporting outcomes such as reductions in the incidence of carbapenem-resistant gram-negative bacilli, adherence to guideline implementations [58], and improvements in rapid diagnosis and treatment initiation [63].

Education and capacity building interventions aimed at increasing knowledge and improving practices related to antimicrobial use. The outcomes measured included knowledge gain [54], training course design and implementation [76], and levels of awareness, attitudes, and intentions to practice antibiotic prescription [85].

Policy and regulation interventions included restrictive measures on antimicrobial sales and commercialization, with outcomes such as reductions in antimicrobial resistance profiles [57, 73] and risk of hospital infections [82].

Awareness interventions aimed at reducing unnecessary antibiotic prescriptions through educational sessions and guideline implementation, with outcomes such as antibiotic prescription rates and appropriateness of prescriptions [75].

### Study design for evaluation studies

The majority of the studies (*n* = 24) used a quasi-experimental design to evaluate interventions. These studies included various types of interventions across multiple sectors. For example, several studies focused on antimicrobial stewardship in human health, implementing strategies such as prescription restrictions, audits, and educational feedback [47–54, 59, 62–64, 69, 70, 74–76, 79, 81, 82, 85].

Observational studies (*n* = 24) were also prevalent among the included studies. These studies often focused on AMS, IPC, and integrated surveillance in human health [56, 58, 60, 61, 65, 68, 77, 78, 80, 83, 84, 87–89].

Only a small subset of studies (*n* = 4) used an experimental design. These studies also spanned various types of interventions like RCT for length of antibiotics use or feeding animals with and without antimicrobials. [45, 55, 66, 73].

## Discussion

In this scoping review of interventions aimed at curbing AMR in the LAC region, we highlight six main findings. First, the majority of studies were concentrated in Latin America, with only a single study originating from the Caribbean region. Second, the studies focused on three primary types of interventions namely, AMS, awareness and education, and IPC. Third, the interventions mainly focused on healthcare providers, with many of these interventions conducted in hospital settings. Fourth, most of the studies were quasi-experimental, only four reported using an experimental design. Fifth, there is wide heterogeneity in the selected outcomes when evaluating the same intervention. Sixth, nearly one-third of the studies did not assessed outcomes or impacts of the interventions.

The concentration of published research from hospital settings related to AMS suggests that current research efforts in the region are skewed towards immediate clinical applications of AMR. Our findings align with the review by Hegewisch-Taylor et al. [11], which also highlighted an increasing trend in AMS initiatives in hospitals. Similarly, the scoping review of national AMS activities by Kamere et al. [13] conducted in eight African countries, identified a predominant focus on human health interventions and significant gaps in the animal and environmental sectors. In line with literature from other regions in the world [97] this focus on AMS and healthcare settings reflects the immediate clinical urgency of AMR, as well as the availability of funding and expertise in these areas. Conversely, the limited number of animal and environmental health intervention research could be due to underfunding and a lower priority of these sectors. The absence of concrete policies, as evidenced in the latest TrACSS report [98], as well as scant published research in the animal and environmental sectors requires further inquiry and detailed analysis of possible barriers to developing policies and conducting research beyond the human health sector.

Several Caribbean countries have reported data in TrACSS, indicating some level of AMR preparedness and action. However, our scoping review found no empirical studies evaluating these efforts in the Caribbean region except for one study from Guadalupe [72]. This absence of published evaluations may be due to limited research output or challenges in accessing region-specific data. A document analysis exploring the AMS policy landscape in Barbados, Guyana, and Saint Lucia [99], identified only 15 relevant records out of 726 initial entries, highlighting the scarcity of accessible information on AMS activities in the Caribbean. This poses a significant gap in our understanding of AMR preparedness and underscores the urgent need to contact individuals involved in these efforts to better understand the scope of the activities and their documentation, especially within the Caribbean.

In terms of the distribution of studies by One Health AMR pillars, studies with and without evaluated interventions were similar, except for a notably higher emphasis on surveillance in studies without evaluated results. The higher focus on integrated surveillance may suggest that while surveillance is recognized as crucial, its evaluation may be more challenging or less prioritized. A scoping review [100] identified barriers such as limited capacity, inadequate data infrastructure, and policy constraints that hinder the effective implementation and utilization of AMR surveillance systems. Additionally, the absence of standardized guidelines and metrics for monitoring and evaluating integrated One Health approaches complicates the assessment of these systems effectiveness. These factors contribute to the observed gap between the recognition of surveillance's importance and the practical challenges in its evaluation. This gap highlights the need for enhanced methodologies and frameworks to assess the impact of surveillance on changing practice or behavior.

Finally, the limited number of studies included in this review can be attributed to two main factors. First, as highlighted in previous reviews, the number of AMR intervention studies conducted in the LAC region is relatively small compared to other regions worldwide [97, 101]. Second, the inclusion criteria for this review were restricted to a narrow time frame, specifically studies published between 2018 and 2024. This narrow time frame was intentionally selected to focus on the most recent interventions implemented and documented in the region, with the aim of informing the prioritization of funding allocations.

## Limitations

This scoping review has several limitations that should be acknowledged. First, our analysis relied entirely on the information reported by the included studies, which introduces a dependence on the accuracy and completeness of their descriptions. This limitation applies broadly, including the classification of study designs, the reporting of intervention activities, and the description of outcomes. The heterogeneity in reporting styles and levels of detail across studies made it challenging to standardize the data and may have resulted in some variability in our categorizations.

Second, the classification of interventions according to the AMR pillars and the sectors involved was based on the information provided in the studies and the interpretation of the reviewers. While efforts were made to apply consistent criteria, this process may have introduced interpretation bias, particularly for studies with vague or incomplete descriptions of their objectives and activities.

Third, although our inclusion criteria did not require explicit mention of AMR as a primary objective, we relied on the introduction or background sections to determine whether interventions were directly or indirectly aimed at addressing AMR. While this approach broadened the scope of included studies, it may still have excluded relevant interventions that did not clearly articulate their connection to AMR.

Fourth, the focus of this review is the bacterial resistance, which was chosen because it poses the most immediate and widespread global public health threat. Emerging resistance to antifungal, antiparasitic and antiviral agents underscores the need for future research to assess interventions targeting these drug classes as well [102, 103].

Finally, while we systematically mapped and described the interventions, study designs, and thematic focuses, this review did not evaluate the quality of the included studies or their reported outcomes. As such, our findings are limited to describing the landscape of AMR-related research and do not provide conclusions regarding the effectiveness or methodological rigor of the interventions.

These limitations highlight the need for future research to conduct quality assessments of individual studies, to refine classification criteria for interventions, and to explore more systematic methods and databases for identifying relevant initiatives with implicit AMR-related objectives and particularly in animal or environmental health. Such efforts will enhance the reliability and comprehensiveness of evidence in this critical area.

## Conclusions

Our findings provide a comprehensive overview of existing AMR interventions in the LAC region, highlighting significant research gaps. The limited evidence on interventions aimed at reducing AMR across One Health sectors—human, animal, and environmental health—poses a challenge for users, prescribers, and decision-makers. Current research is disproportionately focused on clinical applications and human health surveillance, with fewer studies addressing animal health and even fewer exploring environmental health. Most interventions are implemented in healthcare facilities, with limited attention to community settings, animal farms, or environmental contexts.

Based on these results, we strongly recommend that decision-makers prioritize the One Health approach when addressing AMR. It is equally important for this message to reach key stakeholders, including the research community, funders, and the global AMR community, as these groups play a pivotal role in shaping research agendas, allocating resources, and driving comprehensive AMR interventions.

To advance progress, we emphasize the importance of using these results to prioritize funding allocation to the LAC region and to promote increased research across all sectors, with a particular emphasis on animal and environmental health. Besides, rigorous evaluations should accompany these interventions to generate robust, actionable evidence for policy development. Finally, we also advocate for the establishment of an AMR –One health network within the LAC region, to strengthen high-quality research across sectors and facilitate the implementation of effective interventions in countries lagging behind within the region. 

## Supplementary Information

Supplementary Material 1.

## Data Availability

No datasets were generated or analysed during the current study.
