# Supplementary material for: Antimicrobial resistance interventions in Latin America and the Caribbean: a scoping review of reported interventions between 2018–2024
Source: Antimicrob Resist Infect Control. 2025 Nov 11;14:137. doi: 10.1186/s13756-025-01629-z (PMC12607118; doi:10.1186/s13756-025-01629-z)
Supplement: Supplementary file 1 — Supplementary Material 1. [file 13756_2025_1629_MOESM1_ESM.docx]

**Supplementary Material**

**Appendix table 1. Systematic literature search**

Filter: Last 5 years

Date June 29th, 2023. Updated until Dec 31^st^, 2024

**Pubmed**

|  | **Search Texts and Syntaxes** | **Results**  **June 29th, 2023** | **Results updated until**  **Dec 31st, 2024** |
| --- | --- | --- | --- |
| 1: Topic | ((("Drug Resistance, Microbial"[Mesh] OR AMR[tiab] OR biosecurity[tiab] OR "food safety"[tiab] OR "one health"[tiab] OR ((Antibiotic[tiab] OR antimicrobial[tiab] OR "Anti-Infective Agents"[Mesh] OR antibacteria*[tiab] OR "Bacteria"[Mesh] OR Bacteria*[tiab]) AND (Resistance[tiab] OR multiresistance[tiab] OR consumption[tiab] OR use[tiab] OR reduction[tiab] OR restriction[tiab]))) |  |  |
| 2: Intervention | ("infection prevention control"[tiab] OR IPC[tiab] OR stewardship[tiab] OR "Environmental health"[TIAB] OR "animal health"[tiab] OR legislation[tiab] OR regulation[tiab] OR polic*[tiab] OR intervention*[tiab] OR plan*[tiab] OR campaign[tiab] OR program*[tiab] OR strategy[tiab] OR strategies[tiab] implementation[tiab] OR implementing[tiab] OR training[tiab] OR Promotion[tiab] OR initiative[tiab] OR monitor*[tiab] OR "capacity building"[tiab]) |  |  |
| 3: Broader topic | Antimicrobial stewardship[tiab] |  |  |
| 4: Geographical context | ((("aruba"[MeSH Terms] OR "aruba"[tiab]) OR ("argentina"[MeSH Terms] OR "argentina"[tiab]) OR "Antigua and Barbuda"[tiab] OR ("brazil"[MeSH Terms] OR "brazil"[tiab]) OR Brasil[tiab] OR ("bolivia"[MeSH Terms] OR "bolivia"[tiab]) OR "British Virgin Islands"[All Fields] OR ("belize"[MeSH Terms] OR "belize"[tiab]) OR ("barbados"[MeSH Terms] OR "barbados"[tiab]) OR ("bahamas"[MeSH Terms] OR "bahamas"[tiab]) OR ("chile"[MeSH Terms] OR "chile"[tiab]) OR ("cuba"[MeSH Terms] OR "cuba"[tiab]) OR "Costa Rica"[tiab] OR ("colombia"[MeSH Terms] OR "colombia"[tiab]) OR "Cayman Islands"[tiab] OR ("curacao"[MeSH Terms] OR "curacao"[tiab]) OR ("ecuador"[MeSH Terms] OR "ecuador"[tiab]) OR "El Salvador"[tiab] OR "French Guiana"[tiab] OR ("guatemala"[MeSH Terms] OR "guatemala"[tiab]) OR ("guadeloupe"[MeSH Terms] OR "guadeloupe"[tiab]) OR ("haiti"[MeSH Terms] OR "haiti"[tiab]) OR ("haiti"[MeSH Terms] OR "haiti"[tiab]) OR ("guyana"[MeSH Terms] OR "guyana"[tiab]) OR ("grenada"[MeSH Terms] OR "grenada"[tiab]) OR "Dominican Republic"[tiab] OR "Republica Dominicana"[tiab] OR ("dominica"[MeSH Terms] OR "dominica"[tiab]) OR ("honduras"[MeSH Terms] OR "honduras"[tiab]) OR ("jamaica"[MeSH Terms] OR "jamaica"[tiab]) OR ("martinique"[MeSH Terms] OR "martinique"[tiab]) OR ("mexico"[MeSH Terms] OR "mexico"[tiab]) OR ("mexico"[MeSH Terms] OR "mexico"[tiab]) OR ("nicaragua"[MeSH Terms] OR "nicaragua"[tiab]) OR ("paraguay"[MeSH Terms] OR "paraguay"[tiab]) OR ("peru"[MeSH Terms] OR "peru"[tiab]) OR ("panama"[MeSH Terms] OR "panama"[tiab]) OR "Puerto Rico"[tiab] OR "Panama Canal Zone"[tiab] OR "Saint Martin"[tiab] OR "Saint Barthelemy"[tiab] OR "Saint Barthelemy"[tiab] OR ("suriname"[MeSH Terms] OR "suriname"[tiab]) OR "St. Lucia"[tiab] OR "St. Kitts and Nevis"[All Fields] OR "Sint Maarten (Dutch part)"[All Fields] OR "St. Vincent and the Grenadines"[All Fields] OR "St. Martin"[All Fields] OR "Turks and Caicos Islands"[All Fields] OR "Trinidad and Tobago"[All Fields] OR ("uruguay"[MeSH Terms] OR "uruguay"[tiab]) OR ("venezuela"[MeSH Terms] OR "venezuela"[tiab]) OR "Virgin Islands (U.S.)"[All Fields] OR (("venezuela"[MeSH Terms] OR "venezuela"[All Fields]) AND RB[All Fields]) OR "Latin America"[All Fields] OR "Latin American"[All Fields] OR "America Latina"[All Fields] OR "South America"[Mesh] OR "Latin America"[Mesh] OR "Central America"[Mesh] OR "Central American"[All Fields] OR "South American"[All Fields])) |  |  |
| ((#1 AND #2) OR #3) AND #4 | ((("Drug Resistance, Microbial"[Mesh] OR AMR[tiab] OR biosecurity[tiab] OR "food safety"[tiab] OR "one health"[tiab] OR ((Antibiotic[tiab] OR antimicrobial[tiab] OR "Anti-Infective Agents"[Mesh] OR antibacteria*[tiab] OR "Bacteria"[Mesh] OR Bacteria*[tiab]) AND (Resistance[tiab] OR multiresistance[tiab] OR consumption[tiab] OR use[tiab] OR reduction[tiab] OR restriction[tiab]))) AND ("infection prevention control"[tiab] OR IPC[tiab] OR stewardship[tiab] OR "Environmental health"[TIAB] OR "animal health"[tiab] OR legislation[tiab] OR regulation[tiab] OR polic*[tiab] OR intervention*[tiab] OR plan*[tiab] OR campaign[tiab] OR program*[tiab] OR strategy[tiab] OR strategies[tiab] implementation[tiab] OR implementing[tiab] OR training[tiab] OR Promotion[tiab] OR initiative[tiab] OR monitor*[tiab] OR "capacity building"[tiab])) OR Antimicrobial stewardship[tiab]) AND ((("aruba"[MeSH Terms] OR "aruba"[tiab]) OR ("argentina"[MeSH Terms] OR "argentina"[tiab]) OR "Antigua and Barbuda"[tiab] OR ("brazil"[MeSH Terms] OR "brazil"[tiab]) OR Brasil[tiab] OR ("bolivia"[MeSH Terms] OR "bolivia"[tiab]) OR "British Virgin Islands"[All Fields] OR ("belize"[MeSH Terms] OR "belize"[tiab]) OR ("barbados"[MeSH Terms] OR "barbados"[tiab]) OR ("bahamas"[MeSH Terms] OR "bahamas"[tiab]) OR ("chile"[MeSH Terms] OR "chile"[tiab]) OR ("cuba"[MeSH Terms] OR "cuba"[tiab]) OR "Costa Rica"[tiab] OR ("colombia"[MeSH Terms] OR "colombia"[tiab]) OR "Cayman Islands"[tiab] OR ("curacao"[MeSH Terms] OR "curacao"[tiab]) OR ("ecuador"[MeSH Terms] OR "ecuador"[tiab]) OR "El Salvador"[tiab] OR "French Guiana"[tiab] OR ("guatemala"[MeSH Terms] OR "guatemala"[tiab]) OR ("guadeloupe"[MeSH Terms] OR "guadeloupe"[tiab]) OR ("haiti"[MeSH Terms] OR "haiti"[tiab]) OR ("haiti"[MeSH Terms] OR "haiti"[tiab]) OR ("guyana"[MeSH Terms] OR "guyana"[tiab]) OR ("grenada"[MeSH Terms] OR "grenada"[tiab]) OR "Dominican Republic"[tiab] OR "Republica Dominicana"[tiab] OR ("dominica"[MeSH Terms] OR "dominica"[tiab]) OR ("honduras"[MeSH Terms] OR "honduras"[tiab]) OR ("jamaica"[MeSH Terms] OR "jamaica"[tiab]) OR ("martinique"[MeSH Terms] OR "martinique"[tiab]) OR ("mexico"[MeSH Terms] OR "mexico"[tiab]) OR ("mexico"[MeSH Terms] OR "mexico"[tiab]) OR ("nicaragua"[MeSH Terms] OR "nicaragua"[tiab]) OR ("paraguay"[MeSH Terms] OR "paraguay"[tiab]) OR ("peru"[MeSH Terms] OR "peru"[tiab]) OR ("panama"[MeSH Terms] OR "panama"[tiab]) OR "Puerto Rico"[tiab] OR "Panama Canal Zone"[tiab] OR "Saint Martin"[tiab] OR "Saint Barthelemy"[tiab] OR "Saint Barthelemy"[tiab] OR ("suriname"[MeSH Terms] OR "suriname"[tiab]) OR "St. Lucia"[tiab] OR "St. Kitts and Nevis"[All Fields] OR "Sint Maarten (Dutch part)"[All Fields] OR "St. Vincent and the Grenadines"[All Fields] OR "St. Martin"[All Fields] OR "Turks and Caicos Islands"[All Fields] OR "Trinidad and Tobago"[All Fields] OR ("uruguay"[MeSH Terms] OR "uruguay"[tiab]) OR ("venezuela"[MeSH Terms] OR "venezuela"[tiab]) OR "Virgin Islands (U.S.)"[All Fields] OR (("venezuela"[MeSH Terms] OR "venezuela"[All Fields]) AND RB[All Fields]) OR "Latin America"[All Fields] OR "Latin American"[All Fields] OR "America Latina"[All Fields] OR "South America"[Mesh] OR "Latin America"[Mesh] OR "Central America"[Mesh] OR "Central American"[All Fields] OR "South American"[All Fields])**)** | 750 | 308 |

**Web of Science**

|  | **Search Texts and Syntaxes** | **Results** |
| --- | --- | --- |
| 1: Topic | AND (TS=(stewardship OR “one health” OR antibiotic OR antimicrobial OR antiinfective OR antibacteria* OR biosecurity OR &quot;food safety&quot; OR &quot;Anti-Infective Agents&quot; OR Bacteria*)) AND (TS=(Resistance OR multiresistance OR consumption OR use OR restriction OR &quot;Microbial Drug Resistance&quot; OR “Antimicrobial resistance” OR AMR)) |  |
| 2: Intervention | (TS=(“infection prevention control” OR IPC OR legislation OR regulation OR polic* OR intervention* OR plan* OR campaign OR program* OR strategy OR strategies OR implement* OR train* OR Promot* OR initiative OR monitor*)) |  |
| 3: Geographical context | (TS=(&quot;Latin America&quot; OR &quot;America Latina&quot; OR &quot;South America&quot; OR &quot;Central America&quot; OR &quot;South American&quot; OR aruba OR argentina OR Antigua and Barbuda OR brazil OR bolivia OR British Virgin Islands OR belize OR barbados OR bahamas OR chile OR cuba OR Costa Rica OR colombia OR Cayman Islands OR curacao OR ecuador OR El Salvador OR French Guiana OR guatemala OR guadeloupe OR haiti OR guyana OR grenada OR Dominican Republic OR dominica OR honduras OR jamaica OR martinique OR mexico OR nicaragua OR paraguay OR peru OR panama OR Puerto Rico OR Panama Canal Zone OR Saint Martin OR Saint Barthelemy OR suriname OR St. Lucia OR St. Kitts and Nevis OR Sint Maarten OR St. Vincent and the Grenadines OR St. Martin OR Turks and Caicos Islands OR Trinidad and Tobago OR uruguay OR venezuela OR Virgin Islands U.S.)) |  |
| #1 AND #2 AND #3 | (TS=(“infection prevention control” OR IPC OR legislation OR regulation OR polic* OR intervention* OR plan* OR campaign OR program* OR strategy OR strategies OR implement* OR train* OR Promot* OR initiative OR monitor*)) AND (TS=(stewardship OR “one health” OR antibiotic OR antimicrobial OR antiinfective OR antibacteria* OR biosecurity OR &quot;food safety&quot; OR &quot;Anti-Infective Agents&quot; OR Bacteria*)) AND (TS=(Resistance OR multiresistance OR consumption OR use OR restriction OR &quot;Microbial Drug Resistance&quot; OR “Antimicrobial resistance” OR AMR)) AND (TS=(&quot;Latin America&quot; OR &quot;America Latina&quot; OR &quot;South America&quot; OR &quot;Central America&quot; OR &quot;South American&quot; OR aruba OR argentina OR Antigua and Barbuda OR brazil OR bolivia OR British Virgin Islands OR belize OR barbados OR bahamas OR chile OR cuba OR Costa Rica OR colombia OR Cayman Islands OR curacao OR ecuador OR El Salvador OR French Guiana OR guatemala OR guadeloupe OR haiti OR guyana OR grenada OR Dominican Republic OR dominica OR honduras OR jamaica OR martinique OR mexico OR nicaragua OR paraguay OR peru OR panama OR Puerto Rico OR Panama Canal Zone OR Saint Martin OR Saint Barthelemy OR suriname OR St. Lucia OR St. Kitts and Nevis OR Sint Maarten OR St. Vincent and the Grenadines OR St. Martin OR Turks and Caicos Islands OR Trinidad and Tobago OR uruguay OR venezuela OR Virgin Islands U.S.)) | 3689 |

**Lilacs** [**https://lilacs.bvsalud.org/es/**](https://lilacs.bvsalud.org/es/)

**Filter Last 5 years**

|  | **Search Texts and Syntaxes** | **Results** |
| --- | --- | --- |
| 1 | (resistance OR stewardship) AND (antimicrobial OR antibiotic) AND (intervention OR policy OR program OR evaluation OR initiative OR impact) | 274 |
| 2 | (resistencia OR administración) AND (antimicrobiana OR antibiótico) AND (intervención OR política OR programa OR evaluación OR iniciativa OR impacto) | 171 |

**Gray literature**

**Web pages of relevant institutions**

**Filter: Since 2018**

|  | **Relevant institutions** |
| --- | --- |
| 1 | World Health Organization - WHO |
| 2 | Food and Agriculture Organization - FAO |
| 3 | Center for Disease Control and Prevention - CDC, UNEP (Programa de las Naciones Unidas para el Medio Ambiente), Welcome trust / REACT |
| 4 | United Nations Environment Program - UNEP |
| 5 | Welcome Trust |
| 6 | ReAct – Action on Antibiotic Resistance |
| 7 | World Organization for Animal Health - WHOA |
| 8 | International Development Research Centre - IDRC |
| 9 | World Bank |
| 10 | Inter-American Development Bank - IADB |
| 11 | International Centre for Antimicrobial Resistance Solutions - ICARS |
| 12 | U.S. Agency for International Development - USAID |

**Appendix Table 2. Articles excluded and reason for exclusion. Search until Jun 29^th^, 2023.**

|  | **Author, year** | **Title** | **Reason for exclusion** |
| --- | --- | --- | --- |
| 1 | Calero-Cáceres, 2023 | Whole-genome sequencing for surveillance of antimicrobial resistance in Ecuador: present and future implications | Does not describe implementation of a program |
| 2 | Alves, 2019 | Preparation of the discharge of relatives of children using antibiotics: contributions of nursing | Does not describe implementation of a program |
| 3 | Cabral, 2018 | Racionalização de antimicrobianos em ambiente hospitalar | Does not describe implementation of a program |
| 4 | Moore, 2023 | Rapid Diagnostic Test Value and Implementation in Antimicrobial Stewardship Across Low-to-Middle and High-Income Countries: A Mixed-Methods Review | Does not describe implementation of a program |
| 5 | Staneloni, 2022 | Programa de prevención de Enterobacterias Productoras de Carbapenemasas en unidades críticas en Argentina durante la pandemia COVID-19 | Duplicate |
| 6 | Pereira, 2021 | Impact of urinary selective antibiogram in primary care | Wrong country |
| 7 | Rojas-Bonilla, 2020 | Impacto de un programa de optimización de uso de antimicrobianos en un hospital pediátrico de tercer nivel en Panamá | Duplicate |
| 8 | Vegyar, 2020 | Whole-genome sequencing as part of national and international surveillance programmes for antimicrobial resistance: a roadmap | Does not describe implementation of a program |
| 9 | Zhen, 2018 | The impact of prescriptions audit and feedback for antibiotic use in rural clinics: interrupted time series with segmented regression analysis | Wrong country |
| 10 | Genne-Bacon, 2018 | The PARE Project: A Short Course-Based Research Project for National Surveillance of Antibiotic-Resistant Microbes in Environmental Samples | Wrong country |
| 11 | Yoshiaki Gu,2021 | Outcomes and Future Prospect of Japan’s National Action Plan on Antimicrobial Resistance (2016–2020) | Wrong country |
| 12 | Cisneros, 2014 | Global impact of an educational antimicrobial stewardship programme on prescribing practice in a tertiary hospital centre | Wrong country |
| 13 | Vekemans,2021 | Leveraging Vaccines to Reduce Antibiotic Use and Prevent Antimicrobial Resistance: A World Health Organization Action Framework | Wrong country |
| 14 | Departamento de Salud Animal, Chile | Informe sobre uso de antimicrobianos en la salmonicultura nacional Primer Semestre - Año 2023 | Duplicate |
| 15 | WHO, 2023 | Antimicrobial resistance surveillance in Europe 2023–2021 data | Wrong country |
| 16 | WHO, 2022 | Central Asian and European Surveillance of Antimicrobial Resistance: external quality assessment results 2020 | Wrong country |
| 17 | Caipo, 2023 | A qualitative approach for a situation analysis of AMR risks in the food animal production sector | Does not describe implementation of a program |
| 18 | Oliveira Nunes, 2022 | Policy and strategies addressing prevention and control of antimicrobial resistance in Brazil: A scoping review protocol | Does not describe implementation of a program |
| 19 | Sharma, 2021 | Multi-country cross-sectional study of colonization with multidrug-resistant organisms: protocol and methods for the Antibiotic Resistance in Communities and Hospitals (ARCH) studies | Does not describe implementation of a program |
| 20 | Mello, 2021 | Overview of the actions to combat bacterial resistance in large hospitals | Does not describe implementation of a program |
| 21 | Grandon, 2021 | Proposed protocol for performing MIC testing to determine the antimicrobial susceptibility ofRenibacterium salmoninarumin Chilean salmon farms | Does not describe implementation of a program |
| 22 | Cars, 2021 | Resetting the agenda for antibiotic resistance through a health systems perspective | Does not describe implementation of a program |
| 23 | Unemo, 2021 | WHO global antimicrobial resistance surveillance for Neisseria gonorrhoeae 2017-18: a retrospective observational study | Does not describe implementation of a program |
| 24 | Rodriguez, 2020 | Multicenter study of adherence to guidelines on surgical prophylaxis and the determinants of non-adherence in Argentina | Does not describe implementation of a program |
| 25 | Martins, 2019 | Impact of medication therapy management on pharmacotherapy safety in an intensive care unit | Does not describe implementation of an antibiotic program |
| 26 | Alvim, 2019 | O enfermeiro nos programas de gerenciamento do uso de antimicrobianos: revisão integrativa / El enfermero en los programas de optimización del uso de antimicrobianos: revisión integrativa / Participation of nurses in antimicrobial stewardship programs: an integrative review | Does not describe implementation of an antibiotic program |
| 27 | Galarce, 2019 | Se establece una mesa de trabajo intersectorial para enfrentar la resistencia bacteriana en animales peque√±os | Does not describe implementation of a program |
| 28 | Nathwani, 2019 | Value of hospital antimicrobial stewardship programs [ASPs]: a systematic review | Does not describe implementation of a program |
| 29 | Piltcher, 2018 | How to avoid the inappropriate use of antibiotics in upper respiratory tract infections? A position statement from an expert panel | Does not describe implementation of a program |
| 30 | Sawatzky, 2018 | Quality assurance for antimicrobial susceptibility testing of Neisseria gonorrhoeae in Latin American and Caribbean countries, 2013-2015 | Does not describe implementation of a program |
| 31 | Zylbersztajn, 2018 | Therapeutic monitoring of antimicrobial agents in pediatrics. Review based on Latin American experiences | Does not describe implementation of a program |
| 32 | Cruz, 2021 | Resistencia antimicrobiana y factores de virulencia en aislados de Vibrio cholerae O1. Cuba, 2012-2015 | Does not describe implementation of a program |
| 33 | Salgado-Caxito, 2022 | Qualitative Risk Assessment for Antimicrobial Resistance among Humans from Salmon Fillet Consumption Due to the High Use of Antibiotics against Bacterial Infections in Farmed Salmon | Does not describe implementation of a program |
| 34 | Sakeena, 2018 | Non-prescription sales of antimicrobial agents at community pharmacies in developing countries: a systematic review | Does not include articles published in time frame of interest |
| 35 | Yock-Corrales, 2023 | Regional Perspective of Antimicrobial Stewardship Programs in Latin American Pediatric Emergency Departments | Does not describe implementation of a program |
| 36 | Afari-Asiedu, 2022 | Interventions to improve dispensing of antibiotics at the community level in low and middle income countries: a systematic review | Does not describe implementation of a program |
| 37 | Araujo, 2022 | Prevención y control de la resistencia a los antimicrobianos en la Atención Primaria de Salud: evidencia para políticas | Does not include articles published in time frame of interest |
| 38 | Aanensen, 2021 | Implementing Whole-Genome Sequencing for Ongoing Surveillance of Antimicrobial Resistance: Exemplifying Insights Into Klebsiella pneumoniae | Does not describe implementation of a program |
| 39 | Ricieri, 2021 | PRAT tool: a harmonization of antimicrobial stewardship program interventions | Does not describe implementation of a program |
| 40 | Aponte-Gonzalez, 2021 | Preferences based interventions to address the use of antibiotics without prescription: A discrete choice experiment | Does not describe implementation of a program |
| 41 | Lim, 2021 | Surveillance strategies using routine microbiology for antimicrobial resistance in low- and middle-income countries | Does not describe implementation of a program |
| 42 | Donà, 2020 | Implementation and impact of pediatric antimicrobial stewardship programs: a systematic scoping review | Does not include articles published in time frame of interest |
| 43 | Dhingra, 2020 | Microbial Resistance Movements: An Overview of Global Public Health Threats Posed by Antimicrobial Resistance, and How Best to Counter | Does not describe implementation of a program |
| 44 | Manikam, 2020 | Operationalising a One Health approach to reduce the infection and antimicrobial resistance (AMR) burden in under-5-year-old urban slum dwellers: The Childhood Infections and Pollution (CHIP) Consortium | Does not describe implementation of a program |
| 45 | Majumder, 2020 | Tackling Antimicrobial Resistance by promoting Antimicrobial stewardship in Medical and Allied Health Professional Curricula | Does not describe implementation of a program |
| 46 | Silva, 2019 | O Ensino de Gestão de Antimicrobianos em Escola Médica do Rio de Janeiro | Does not describe implementation of a program |
| 47 | Cabrera-Pardo, 2019 | A One Health - One World initiative to control antibiotic resistance: A Chile - Sweden collaboration | Does not describe implementation of a program |
| 48 | Sampaio, 2018 | Implementação da nova regulamentação para prescrição e dispensação de antimicrobianos: possibilidades e desafios | Does not describe implementation of a program |
| 49 | Millanao, 2018 | Resistencia a los antimicrobianos en Chile y el paradigma de Una Salud: manejando los riesgos para la salud pública humana y animal resultante del uso de antimicrobianos en la acuicultura del salmón y en medicina | Does not describe implementation of a program |
| 50 | Pardo, 2018 | ["One health" approach in the actions to address antimicrobial resistance from a latin american standpoint] | Does not describe implementation of a program |
| 51 | Aguiar, 2023 | [The evolution of Brazilian human health policies for the prevention and control of antimicrobial resistance: a scoping reviewEvolución de las políticas de prevención y control de la resistencia a los antimicrobianos desde la perspectiva de la salud humana en Brasil: revisión exploratoria] | Does not describe implementation of a program |
| 52 | WHO, 2020 | Antimicrobial stewardship programmes in health-care facilities in low- and middle-income countries: a WHO practical toolkit | Does not describe implementation of a program, describes a toolkit |
| 53 | Sevilha-Santos, 2022 | Sensitivity of different DNA extraction methods and PCR to detect resistance in patients with leprosy stratified by the bacilloscopic index | Does not describe implementation of a program |
| 54 | Fuga, 2022 | WHO Critical Priority Escherichia coli as One Health Challenge for a Post-Pandemic Scenario: GeNomic Surveillance and Analysis of Current Trends in Brazil | Describes results for a Surveillance study, but not a surveillance program |
| 55 | Carvalhaes, 2023 | Performance of the Vitek 2 Advanced Expert System (AES) as a Rapid Tool for Reporting Antimicrobial Susceptibility Testing (AST) in Enterobacterales from North and Latin America | Describes results for a Surveillance study, but not a surveillance program |
| 56 | Moore, 2023 | Rapid DiagNostic Test Value and Implementation in Antimicrobial Stewardship Across Low-to-Middle and High-Income Countries: A Mixed-Methods Review | Duplicate |
| 57 | Vaucher, 2022 | Patient safety program focused on antimicrobial stewardship strategies for Staphylococcus spp | Does not describe implementation of a program |
| 58 | Ministerio de Saude de Brazil, 2019 | Plano de Ação Nacional de Prevenção e Controle da Resistência aos Antimicrobianos no Âmbito da Saúde Única Ministério da Saúde Brasília DF 2019 | Describes a National Action plan, but not a particular intervention |
| 59 | Garza-González, 2019 | A snapshot of antimicrobial resistance in Mexico. Results from 47 centers from 20 states during a six-month period | Describes the results of a prevalence study. It does not describe an intervention or program. |
| 60 | MINSALUD Chile, 2018 | Plan Nacional de Respuesta a la Resistencia a los AntimicrobiaNos-Plan estratégico | Describes a National Action plan, but not a particular intervention |
| 61 | Hernández-Gámez, 2019 | Programas de optimización del uso de antimicrobianos en Perú: un acuerdo sobre lo fundamental | Duplicate |
| 62 | Naylor, 2023 | A global view of aquaculture policy | Does not describe implementation of a program |
| 63 | Mello, 2019 | Ações para a prevenção e controle da resistência bacteriana em hospitais de grande porte de Minas Gerais | It is a thesis; it does not describe an intervention being o having been implemented |
| 64 | Lob, 2018 | Activity of Ertapenem against Enterobacteriaceae in seven global regions-SMART 2012-2016 | Does not describe implementation of a program |
| 65 | Fabbiani, 2021 | Adecuación al flujograma PROA de neumonía aguda comunitaria en la emergencia del Hospital de Clínicas en 2019, Uruguay | Does not describe implementation of a program |
| 66 | Fuentes, 2021 | Administration and Therapeutic Drug Monitoring of ßlactams and Vancomycin in Critical Care Units in Colombia: The ANTIBIOCOL Study | Does not describe implementation of a program |
| 67 | Aldana, 2021 | Agrochemical leaching reduction in biochar-amended tropical soils of Belize | Describe an experiment, but do not describe the implementation of an intervention |
| 68 | Santos, 2019 | Analise custo-efetividade de antimicrobianos usados em pacientes infectados por Klebsiella Pneumoniae Carbapenemase | It is a thesis and describes an economical evaluation |
| 69 | Guzmán-Terán, 2018 | Análisis de usos y resistencia a antibióticos en una UCI de Montería, Colombia | Describe the state of art of antibiotics consumption in a hospital |
| 70 | Ibarra, 2018 | Animal production, animal health and food safety: Gaps and challenges in the chilean industry | Does not describe implementation of a program |
| 71 | Dominguez, 2021 | Anthropogenic Activities and the Problem of Antibiotic Resistance in Latin America: A Water Issue | Does not describe implementation of a program |
| 72 | Castro, 2020 | Antibiotic consumption in developing countries defies global commitments: an overview on Brazilian growth in consumption | Does not describe implementation of a program |
| 73 | Amabile-Cuevas, 2021 | Antibiotic usage and resistance in Mexico: an update after a decade of change | Does not describe implementation of a program |
| 74 | Sartelli, 2020 | Antibiotic Use in Low and Middle-Income Countries and the Challenges of Antimicrobial Resistance in Surgery | Does not describe implementation of a program |
| 75 | Tenea, 2021 | Antimicrobial Cocktail Combining Specific Peptide Extracts from Native Probiotic Bacteria Hamper Adulteration of Ready-to-Eat Mango Wedges | Does not describe implementation of a program |
| 76 | Avraam, 2021 | Antimicrobial Resistance and Livestock Trade for Low and Middle Income Countries: Regional Analysis of Global Coordination Policies | Describe hypothetical scenarios for AMR policies |
| 77 | Millanao, 2018 | Antimicrobial resistance in Chile and The One Health paradigm: Dealing with threats to human and veterinary health resulting from antimicrobial use in salmon aquaculture and the clinic | Does not describe implementation of a program |
| 78 | Medina-Pizzali, 2021 | Antimicrobial Resistance in Rural Settings in Latin America: A Scoping Review with a One Health Lens | It is a review but does not contain articles describing an AMR intervention during the time frame |
| 79 | Giono-Cerezo, 2020 | Antimicrobial resistance. Its importance and efforts to control | Does not describe implementation of a program, it mentions a national action plan |
| 80 | Hegewisch-Taylor, 2020 | Antimicrobial stewardship in hospitals in Latin America and the Caribbean: a scoping review | It is a review but does not contain articles describing an AMR intervention during the time frame |
| 81 | Fabre, 2022 | Antimicrobial stewardship in Latin America: Past, present, and future | It is a review but does not contain articles describing an AMR intervention during the time frame |
| 82 | Gazal, 2020 | Antimicrobials and resistant bacteria in global fish farming and the possible risk for public health | Wrong country |
| 83 | Pantoja, 2020 | Aplicação de tecnologia educativa na sensibilização do protocolo de sepse em unidade de tocoginecologia | It does mention an intervention for adherence to sepsis guideline, but does not mention anything about antimicrobial resistance |
| 84 | Tayel, 2018 | Application of Quercus infectoria extract as a natural antimicrobial agent for chicken egg decontamination | It describes an experiment that hypothesized a natural product as antimicrobial agent |
| 85 | Duber, 2018 | Appropriate and timely antibiotic administration for neonatal sepsis in Mesoamerica | It describes guideline for neonatal sepsis, but not intendet to fight AMR |
| 86 | Horak, 2019 | Assessing effects of confined animal production systems on water quality, ecological integrity, and macroinvertebrates at small piedmont streams (Patagonia, Argentina) | It does not mention AMR |
| 87 | Farghaly, 2021 | Assessing Knowledge, Practices, Use and Raising Awareness of Antibiotic and Antibiotic Resistance Among Dental Patients Before and After Educational Sessions | Wrong country |
| 88 | Rhea, 2023 | Assessing Livestock Production Practices on Small-Scale Multi-Species Farms Located on Floreana Island, GalÃ¡pagos Islands | It reports on the characterization of animal welfare |
| 89 | Jacobs, 2019 | Assessing the impact of law enforcement to reduce over-the-counter (OTC) sales of antibiotics in low- and middle-income countries; a systematic literature review | It is a review but does not contain articles describing an AMR intervention during the time frame |
| 90 | Souza, 2019 | Avaliação da concordância da dispensação de antimicrobianos em relação aos microrganismos isolados em culturas de um hospital no meio-oeste catarinense | Does not describe implementation of a program |
| 91 | Pinto Jimenez, 2023 | Awareness of antibiotic resistance: a tool for measurement among human and animal health care professionals in LMICs and UMICs | Does not describe implementation of a program |
| 92 | Prince-Guerra, 2020 | Both Handwashing and an Alcohol-Based Hand Sanitizer Intervention Reduce Soil and Microbial Contamination on Farmworker Hands during Harvest, but Produce Type Matters | It does not mention AMR explicitly |
| 93 | Garay, 2021 | Budget impact analysis of using procalcitonin to optimize antimicrobial treatment for patients with suspected sepsis in the intensive care unit and hospitalized lower respiratory tract infections in Argentina | It is an economic evaluation of the potential of an AMR intervention not yet being implemented |
| 94 | de Almeida, 2022 | Community dog program in five municipalities of Parana, Brazil | It does not mention AMR |
| 95 | Romero Viamonte, 2021 | Compliance with antibiotic prophylaxis guidelines in caesarean delivery: a retrospective, drug utilization study (indication-prescription type) at an Ecuadorian hospital | Does not describe implementation of a program |
| 96 | Costa and G, 2020 | Comprehensive evaluation and implementation of improvement actions in bovine abattoirs to reduce pathogens exposure | It does not mention AMR |
| 97 | Oñate, 2021 | Consensus Recommendations Based on Evidence for Abdominal Sepsis in the Pediatric and Adult Population of Colombia | It is not an intervention it is a guideline |
| 98 | Vicente, 2021 | Creating a platform to enable collaborative learning in One Health: The Joint Initiative for Teaching and Learning on Global Health Challenges and One Health experience | It does not mention AMR |
| 99 | Gruel, 2021 | Critical Evaluation of Cross-Sectoral Collaborations to Inform the Implementation of the "One Health" Approach in Guadeloupe | It does not mention AMR, it mentiones other interventions on virus or fungus |
| 100 | Castiblanco, 2019 | Cuidados de enfermería para reducir infecciones por microorganismos oportunistas en pacientes oncológicos | It does not mention AMR |
| 101 | Sato, 2021 | Current Status of Antimicrobial Stewardship Programs in São Paulo Hospitals | Does not describe implementation of a program |
| 102 | Rocha, 2019 | Custo-efetividade de teste rápido de detecção de Klebsiella spp. para rastreio hospitalar | Does not describe implementation of a program, it is an economic evaluation of a diagnostic test |
| 103 | Sneddon, 2018 | Development and impact of a massive open online course (MOOC) for antimicrobial stewardship | Does not describe implementation of a program |
| 104 | Araújo, 2018 | Development of a Checklist for Assessing Good Hygiene Practices of Fresh-Cut Fruits and Vegetables Using Focus Group Interviews | it does not mention AMR specifically |
| 105 | Mastrochirico-Filho, 2020 | Development of a SNP linkage map and genome-wide association study for resistance to Aeromonas hydrophila in pacu (Piaractus mesopotamicus) | Does not describe implementation of a program |
| 106 | Rodrigues, 2023 | Development of an Educational Gamification Strategy to Enhance the Food Safety Practices of Family Farmers in Public Food Markets of Northeast Brazil: A Case Study | it does not mention AMR specifically |
| 107 | El Omeiri, 2023 | Driving multisectoral antimicrobial resistance action in South America: Lessons learned from implementing an enhanced tripartite AMR country self-assessment tool | Does not describe implementation of a program |
| 108 | Sosa-Hernandez, 2021 | Economic and Epidemiological Impact of an Improvement Plan for the Decrease of Ventilator-Associated Pneumonia in a Tertiary Hospital in Mexico | It mentions an intervention to prevent infection, but does not mentioned AMR specifically |
| 109 | Saweri, 2021 | Economic evaluation of point-of-care testing and treatment for sexually transmitted and genital infections in pregnancy in low- and middle-income countries: A systematic review | It is a review but does not contain articles describing an AMR intervention during the time frame |
| 110 | Pettan-Brewer, 2022 | Editorial: Challenges and successes of One Health in the context of planetary health in Latin America and the Caribbean | Does not describe implementation of a program |
| 111 | Galindez, 2018 | Educational strategy to prevent occupational accidents by sharp objects in nurses of a public hospital in venezuela | Does not describe implementation of a program |
| 112 | Camargos, 2021 | Effect of pneumococcal conjugate vaccines on invasive pneumococcal disease | Does not mention AMR specifically |
| 113 | Ormea, 2018 | El enfoque de Una Salud en Perú | Does not describe implementation of a program |
| 114 | Barrantes, 2022 | El impacto de la resistencia a los antibióticos en el desarrollo sostenible | It is a review that leads to national action plan of Costa Rica |
| 115 | Bugueno-Carrasco, 2021 | Elimination of pharmaceutical pollutants by solar photoelectro-Fenton process in a pilot plant | It is a pilot study, basic science, not an implemented intervention |
| 116 | Pardo, 2018 | Enfoque de una salud en las acciones para enfrentar la resistencia a los antimicrobianos desde una Ã³ptica latinoamericana | Does not describe implementation of a program |
| 117 | Cunha, 2020 | Estratégias para o uso seguro de antimicrobianos pela enfermagem no ambiente hospitalar: revisão integrativa | Wrong country |
| 118 | Ospina-García, 2022 | Evaluación de adherencia a la guía de práctica clínica para el manejo de faringoamigdalitis aguda en la atención de urgencias del Hospital Universitario San Ignacio, Bogotá, Colombia | Does not describe implementation of a program |
| 119 | Martínez-Martínez, 2019 | Evaluación del control de infecciones poscesárea mediante la implementación de un programa preventivo | Does not mentioned specifically AMR |
| 120 | Dávila Morán, 2022 | Evaluación microbiológica del caudal de impulsión y extracción mecánica en habitaciones hospitalarias de aislados infecciosos e inmunodeprimidos | Does not mentioned specifically AMR |
| 121 | Signorini, 2018 | Evaluation of decontamination efficacy of commonly used antimicrobial interventions for beef carcasses against Shiga toxin-producing Escherichia coli | Does not describe implementation of a program |
| 122 | Pertile, 2022 | Evaluation of the impact of chemical control on the ecology of Rattus norvegicus of an urban community in Salvador, Brazil | Does not mentioned specifically AMR |
| 123 | Gagetti, 2020 | Evolution of the performance of Latin America Reference Laboratories in the detection of mechanisms of antimicrobial resistance | Does not describe implementation of a program |
| 124 | Cassiolato, 2018 | Expansion of the multidrug-resistant clonal complex 320 among invasive Streptococcus pneumoniae serotype 19A after the introduction of a ten-valent pneumococcal conjugate vaccine in Brazil | Describes a situation not a program |
| 125 | Merigueti, 2019 | FindTargetsWEB: A User-Friendly Tool for Identification of Potential Therapeutic Targets in Metabolic Networks of Bacteria | Does not describe implementation of a program |
| 126 | Ferreira, 2020 | Four levels of evaluation nurse training program on regional food | Does not mentioned specifically AMR |
| 127 | Pettan-Brewer, 2021 | From the Approach to the Concept: One Health in Latin America-Experiences and Perspectives in Brazil, Chile, and Colombia | Does not mentioned specifically AMR |
| 128 | Lazure, 2022 | Gaps and barriers in the implementation and functioning of antimicrobial stewardship programs: results from an educational and behavioral mixed method needs assessment in France, the United States, Mexico and India | Describe the knowledge about AMR in healthcare providers |
| 129 | Urbiztondo, 2018 | General Practitioners' Views on the Acceptability and Applicability of Using Quality Indicators as an Intervention to Reduce Unnecessary Prescription of Antibiotics in Four South American Countries | Does not describe implementation of a program. It describes views and opinions of Healthcare providers on quality indicators for antibiotic use |
| 130 | Lee, 2022 | Geographic patterns of global isolates of carbapenem-resistant Klebsiella pneumoniae and the activity of ceftazidime/avibactam, meropenem/vaborbactam, and comparators against these isolates: Results from the Antimicrobial Testing Leadership and Surveillance (ATLAS) program, 2020 | Does not included enough information about program, describes intervention but not the countries |
| 131 | Melo, 2020 | Gestão das intervenções de prevenção e controle da resistência a antimicrobianos em hospitais: revisão de evidências | Does not included enough information about program, describes intervention but not the countries |
| 132 | Zhou, 2022 | Global antimicrobial resistance: a system-wide comprehensive investigation using the Global One Health Index | Describe the development of a tool, not an intervention being implemented |
| 133 | Sweileh, 2021 | Global research activity on antimicrobial resistance in food-producing animals | Does not describe implementation of a program |
| 134 | Goff, 2022 | Global resilience and new strategies needed for antimicrobial stewardship during the COVID-19 pandemic and beyond | Does not included enough information about program, describes intervention but not the countries |
| 135 | Wade, 2021 | Healthcare-associated infections and the prescribing of antibiotics in hospitalized patients of the Caribbean Community (CARICOM) states: a mixed-methods systematic review | It is a review but does not contain articles describing an AMR intervention during the time frame |
| 136 | Fachini, 2021 | The 4-Year Experience with Implementation and Routine Use of Pathogen Reduction in a Brazilian Hospital | Does not mentioned specifically AMR |
| 137 | Leandro, 2021 | The adoption of the One Health approach to improve surveillance of venomous animal injury, vector-borne and zoonotic diseases in Foz do Iguaçu, Brazil | Does not mentioned specifically AMR |
| 138 | Silva, 2020 | The direct and indirect effects of the pneumococcal conjugated vaccine on carriage rates in children aged younger than 5 years in Latin America and the Caribbean: a systematic review | It is a review but does not contain articles describing an AMR intervention during the time frame |
| 139 | Agudelo, 2021 | The direct effect of pneumococcal conjugate vaccines on invasive pneumococcal disease in children in the Latin American and Caribbean region (SIREVA 2006-17): a multicentre, retrospective observational study | Does not mentioned specifically AMR |
| 140 | Aguiar, 2023 | The evolution of Brazilian human health policies for the prevention and control of antimicrobial resistance: a scoping review | Does not describe implementation of a program |
| 141 | Bae, 2019 | Activity of ceftaroline against pathogens associated with community-acquired pneumonia collected as part of the AWARE surveillance program, 2015–2016 | Does not describe implementation of a program |
| 142 | Beirão, 2020 | Activity of ceftolozane-tazobactam and comparators against gram-negative bacilli: Results from the study for monitoring antimicrobial resistance trends (SMART - Brazil; 2016-2017) | Does not describe implementation of a program |

Abbreviations: AMR (Antimicrobial Resistance), LMICs (Low- and Middle-Income Countries) and UMICs (Upper-Middle-Income Countries)

**Appendix Table 3. Articles excluded and reason for exclusion. Search from Jul, 2023 until Dec, 31st, 2024.**

|  | **Author, year** | **Title** | **Reason for exclusion** |
| --- | --- | --- | --- |
| 1 | Andrade Fernandes, 2024 | The Brazilian collaborative on antimicrobial stewardship: A value-based healthcare approach. | Does not describe implementation of a program |
| 2 | Pallares, 2023 | Antimicrobial stewardship programs in seven Latin American countries: facing the challenges. | Duplicate |
| 3 | Restrepo-Arbeláez, 2023 | Antimicrobial Stewardship Programs in Latin America and the Caribbean: A Story of Perseverance, Challenges, and Goals. | It is a review but do not describe new programs |
| 4 | Rocke, 2023 | READ-ing antimicrobial stewardship in the Caribbean: a tri-nation document review. | Describe an descriptive study, but do not describe the implementation of an intervention |
| 5 | Fabre, 2024 | Knowledge, attitudes and perceptions of Latin American healthcare workers relating to antibiotic stewardship and antibiotic use: a cross-sectional multi-country study. | Describe the development of a tool, not an intervention being implemented |
| 6 | Pasek, 2024 | Vancomycin AUC-Dosing Initiative at a Regional Antibiotic Stewardship Collaborative. | Wrong country |
| 7 | Giron Camerini, 2024 | Nursing strategies in antimicrobial stewardship in the hospital environment: a qualitative systematic review. | It is a review but does not contain articles describing an AMR intervention during the time frame |
| 8 | Fabre, 2024 | Antibiotic Use in Medical-Surgical Intensive Care Units and General Wards in Latin American Hospitals. | Describe the state of art of antibiotics consumption in a hospital |
| 9 | Avello, 2024 | National action plans on antimicrobial resistance in Latin America: an analysis via a governance framework. | Describes a National Action plan, but not a particular intervention |
| 10 | Fabre, 2023 | Deep Dive Into Gaps and Barriers to Implementation of Antimicrobial Stewardship Programs in Hospitals in Latin America. | Does not describe implementation of a program |
| 11 | Martins da Silva, 2024 | Evaluation of the perspective of pharmacists on antimicrobial stewardship and the barriers to its consolidation in hospitals: a cross-sectional study. | Does not describe implementation of a program |
| 12 | Fernandes Ramos, 2023 | Low utilization of vancomycin in febrile neutropenia: real-world evidence from 4 Brazilian centers. | Does not describe implementation of an antibiotic program |
| 13 | Ribeiro Machado, 2024 | Analysis of omission of antimicrobial doses in Intensive Care Units. | Does not describe implementation of an antibiotic program |
| 14 | Lemos Hinrichsen, 2024 | Assessing antimicrobial stewardship governance in Northeast Brazilian hospitals: a survey-based analysis. | Does not describe implementation of a program. It describes views and opinions of Healthcare providers on quality indicators for antibiotic use |
| 15 | Aporta Marins, 2024 | Evaluation of interventions led by pharmacists in antimicrobial stewardship programs in low- and middle-income countries: a systematic literature review. | systematic review whose articles are included |
| 16 | Yeager, 2024 | Phage therapy: resurrecting a historical solution for the contemporary challenge of rising antibiotic resistance in Latin America. | Describe an experiment, but do not describe the implementation of an intervention |
| 17 | Castillo Bejarano, 2024 | Antimicrobial stewardship programs in a Mexican private healthcare system: a self-assessment of core elements. | Does not describe implementation of an antibiotic program |
| 18 | Allel, 2024 | Opportunities and challenges in antimicrobial resistance policy including animal production systems and humans across stakeholders in Argentina: a context and qualitative analysis. | It is a review but does not contain articles describing an AMR intervention during the time frame |
| 19 | De La Cadena, 2023 | [Update of antimicrobial resistance in level III and IV health institutions in Colombia between January 2018 and December 2021]. | Describes the results of a prevalence study. It does not describe an intervention or program. |
| 20 | Bhering,2024 | Strengthening Multidrug-Resistant Tuberculosis Epidemiological Surveillance in Rio de Janeiro: a multidimensional analysis. | Describes the results of a prevalence study. It does not describe an intervention or program. |
| 21 | Garza-González, 2024 | Profile of bacterial resistance in emergency wards, non-intensive care unit areas, and intensive care units in Mexican hospitals with the antimicrobial stewardship program. | Describes the results of a prevalence study. It does not describe an intervention or program. |
| 22 | Morales Taborda, 2024 | Understanding antibiotic knowledge, attitudes, and practices: a cross-sectional study in physicians from a Colombian region, 2023. | Describes a situation not a program |
| 23 | Lazure, 2023 | Gaps and barriers in the implementation and functioning of antimicrobial stewardship programmes: results from an educational and behavioural mixed-methods needs assessment in France, the United States, Mexico and India-authors' response. | Duplicate |
